# Supplementary material for: I wanna hold your hand: Handholding is preferred over gentle stroking for emotion regulation
Source: PLoS One. 2023 Apr 6;18(4):e0284161. doi: 10.1371/journal.pone.0284161 (PMC10079127; doi:10.1371/journal.pone.0284161)
Supplement: S1 Appendix — (DOCX) [file pone.0284161.s001.docx]

**I Wanna Hold Your Hand: Handholding is Subjectively Preferred to Stroking in Everyday Situations**

**Supplementary Material**

**Manipulation Checks**

For all three datasets, we conducted mixed linear analyses to examine whether situations coded as intense were indeed rated as intense and situations coded as positive were indeed rated as positive. To this end, we predicted the ratings using the dichotomous classifications. As expected, dichotomous classifications were indeed associated with ratings in all studies, both for intensity and for positivity (Study 1: b = .821(SE = .048), t(692) = 17.13, p < .001; Study 2: b = .801(SE = .037), t(1514) = 21.851, p < .001; Study 3: b = 1.48(SE = .095), t(101) = 15.614, p < .001); Study 4: b = 0.773(SE = 0.115), t(82)=6.706, p < .001) (Study 1: b = 3.879(SE = .061), t(692) = 63.9, p < .001; Study 2: b = 3.521(SE = .051), t(1514) = 68.373, p < .001). Studies 3 and 4 had no situation positivity classification.

**Valence and Physicality**

Three patterns emerged from the examination of situation valence and physicality as possible moderators of touch preference. In most studies, touch type and physicality interacted, such that handholding was found to be preferable to stroking in emotional situations more than in physical situations. Both types of touch were perceived as more helpful in emotional situations than in physical situations and in negative situations than in positive situations. Other results were inconsistent. Full analysis tables are provided in Tables S1 (ratings) and S2 (dichotomous classifications).

## Dichotomous Intensity and Valence Ratings

When preforming analyses using dichotomous situation classifications instead of ratings (except for the first part of Study 1, analyses for which are provided in the main text), dichotomous classifications were dummy coded 0.5 and -0.5 (to allow for the interpretation of effects as effects that average over categories of other values). Even though the use of repeated measures ANOVA was theoretically possible, we used linear mixed models to preserve equivalence with analyses using the ratings. Still, to test robustness, we performed ANOVA analyses wherever possible; no meaningful difference in results was found.

**Table S1.** The effect of physicality (physical\emotional) as well as situation intensity and valence measured by participant ratings, and touch type on touch ratings. Note that Study 3 did not distinguish situation physicality, and that Study 4 did not distinguish situation valence.

|  |  | b(SE) | 95% CI | t(df) | p | f^2^ |
| --- | --- | --- | --- | --- | --- | --- |
| Study 1 | Intercept | 3.752(.057) | 3.64,3.86 | 65.64(1470) | < .001*** | 0 |
|  | Intensity | 0.075(.024) | 0.03,0.12 | 3.101(1470) | .002** | .004 |
|  | Touch Type | 0.221(.038) | 0.15,0.30 | 5.874(1470) | < .001*** | .014 |
|  | Valence | -0.033(.009) | -0.05,-0.02 | -3.63(1470) | < .001*** | .007 |
|  | Physicality | -0.028(.038) | -0.10,0.05 | -0.736(1470) | .462 | 0 |
|  | Intensity*Touch Type | 0.151(.048) | 0.06,0.25 | 3.129(1470) | .002** | .004 |
|  | Intensity*Valence | -0.015(.012) | -0.04,0.01 | -1.229(1470) | .219 | 0 |
|  | Touch Type*Valence | 0.04(.018) | 0.00,0.08 | 2.199(1470) | .028* | .002 |
|  | Intensity*Physicality | -0.042(.052) | -0.14,0.06 | -0.81(1470) | .418 | .002 |
|  | Touch Type*Physicality | -0.21(.075) | -0.36,-0.06 | -2.788(1470) | .005** | .003 |
|  | Valence*Physicality | -0.041(.018) | -0.08,0.00 | -2.207(1470) | .027* | .004 |
|  | Intensity*Touch Type*Valence | -0.002(.023) | -0.05,0.04 | -0.097(1470) | .922 | 0 |
|  | Intensity*Touch Type*Physicality | 0.03(.096) | -0.16,0.22 | 0.31(1470) | .757 | 0 |
|  | Intensity*Valence*Physicality | 0.045(.024) | 0.00,0.09 | 1.853(1470) | .064^†^ | 0 |
|  | Touch Type*Valence*Physicality | 0.023(.036) | -0.05,0.09 | 0.634(1470) | .526 | 0 |
|  | Intensity*Touch Type*Valence*Physicality | -0.062(.045) | -0.15,0.03 | -1.36(1470) | .174 | .001 |
| Study 2 touch provision | Intercept | 3.874(.067) | 3.74,4.00 | 58.188(2308) | < .001*** | 0 |
|  | Intensity | 0.085(.021) | 0.04,0.13 | 4.034(2308) | < .001*** | .004 |
|  | Touch Type | 0.353(.033) | 0.29,0.42 | 10.654(2308) | < .001*** | .026 |
|  | Valence | -0.011(.009) | -0.03,0.01 | -1.255(2308) | .210 | .001 |
|  | Physicality | -0.113(.033) | -0.18,-0.05 | -3.379(2308) | .001*** | .003 |
|  | Intensity*Touch Type | 0.096(.042) | 0.01,0.18 | 2.28(2308) | .023* | .001 |
|  | Intensity*Valence | -0.012(.011) | -0.03,0.01 | -1.12(2308) | .263 | < 0 |
|  | Touch Type*Valence | -0.002(.018) | -0.04,0.03 | -0.118(2308) | .906 | 0 |
|  | Intensity*Physicality | -0.083(.044) | -0.17,0.00 | -1.903(2308) | .057? | .002 |
|  | Touch Type*Physicality | -0.14(.066) | -0.27,-0.01 | -2.114(2308) | .035* | .001 |
|  | Valence*Physicality | -0.034(.018) | -0.07,0.00 | -1.903(2308) | .057^†^ | .001 |
|  | Intensity*Touch Type*Valence | -0.054(.021) | -0.09,-0.01 | -2.605(2308) | .009** | .002 |
|  | Intensity*Touch Type*Physicality | -0.163(.084) | -0.33,0.00 | -1.939(2308) | .053^†^ | .001 |
|  | Intensity*Valence*Physicality | -0.017(.022) | -0.06,0.03 | -0.786(2308) | .432 | 0 |
|  | Touch Type*Valence*Physicality | -0.004(.035) | -0.07,0.07 | -0.106(2308) | .915 | 0 |
|  | Intensity*Touch Type*Valence*Physicality | -0.047(.041) | -0.13,0.03 | -1.14(2308) | .255 | 0 |
| Study 2 touch reception | Intercept | 3.873(.067) | 3.74,4.00 | 57.637(2308) | < .001*** | 0 |
|  | Intensity | 0.023(.021) | -0.02,0.06 | 1.064(2308) | .287 | 0 |
|  | Touch Type | 0.484(.035) | 0.42,0.55 | 13.896(2308) | < .001*** | .046 |
|  | Valence | -0.04(.009) | -0.06,-0.02 | -4.613(2308) | < .001*** | .006 |
|  | Physicality | -0.021(.035) | -0.09,0.05 | -0.606(2308) | .545 | < 0 |
|  | Intensity*Touch Type | 0.095(.042) | 0.01,0.18 | 2.235(2308) | .026* | .001 |
|  | Intensity*Valence | -0.01(.011) | -0.03,0.01 | -0.929(2308) | .353 | < 0 |
|  | Touch Type*Valence | 0.001(.017) | -0.03,0.03 | 0.043(2308) | .965 | 0 |
|  | Intensity*Physicality | -0.059(.045) | -0.15,0.03 | -1.317(2308) | .188 | 0 |
|  | Touch Type*Physicality | -0.142(.07) | -0.28,-0.01 | -2.033(2308) | .042* | .001 |
|  | Valence*Physicality | -0.043(.018) | -0.08,-0.01 | -2.449(2308) | .014* | .002 |
|  | Intensity*Touch Type*Valence | -0.02(.02) | -0.06,0.02 | -0.985(2308) | .325 | 0 |
|  | Intensity*Touch Type*Physicality | 0.004(.085) | -0.16,0.17 | 0.049(2308) | .961 | 0 |
|  | Intensity*Valence*Physicality | -0.029(.021) | -0.07,0.01 | -1.392(2308) | .164 | < 0 |
|  | Touch Type*Valence*Physicality | -0.008(.035) | -0.08,0.06 | -0.238(2308) | .812 | 0 |
|  | Intensity*Touch Type*Valence*Physicality | 0.003(.041) | -0.08,0.08 | 0.076(2308) | .939 | 0 |
| Study 3 | Intercept | 3.623(.122) | 3.38,3.86 | 29.628(401) | < .001*** | .014 |
|  | Intensity | 0.123(.081) | -0.04,0.28 | 1.527(401) | .128 | .001 |
|  | Touch Type | 1.083(.113) | 0.86,1.31 | 9.559(401) | < .001*** | .122 |
|  | Valence | -0.066(.066) | -0.20,0.06 | -0.998(401) | .319 | 0 |
|  | Intensity*Touch Type | 0.213(.161) | -0.10,0.53 | 1.324(401) | .186 | .002 |
|  | Intensity*Valence | 0.028(.059) | -0.09,0.14 | 0.473(401) | .636 | .004 |
|  | Touch Type*Valence | -0.032(.132) | -0.29,0.23 | -0.239(401) | .811 | 0 |
|  | Intensity*Touch Type*Valence | 0.02(.09) | -0.16,0.20 | 0.217(401) | .828 | 0 |
| Study 4 | Intercept | 53.105(4.722) | 43.79,62.42 | 11.247(181) | < .001*** | .004 |
|  | Intensity | 5.666(2.616) | 0.50,10.83 | 2.165(181) | .032* | < 0 |
|  | Touch Type | 22.39(3.366) | 15.75,29.03 | 6.652(181) | < .001*** | .095 |
|  | Physicality | -13.194(3.478) | -20.06,-6.33 | -3.794(181) | < .001*** | .037 |
|  | Intensity*Touch Type | 2.758(4.942) | -6.99,12.51 | 0.558(181) | .577 | .001 |
|  | Intensity*Physicality | -11.044(6.485) | -23.84,1.75 | -1.703(181) | .090^†^ | < 0 |
|  | Touch Type*Physicality | 5.42(6.732) | -7.86,18.70 | 0.805(181) | .422 | .001 |
|  | Intensity*Touch Type*Physicality | -10.945(9.884) | -30.45,8.56 | -1.107(181) | .270 | .003 |

Note – High valence values refer to situations being perceived as positive.

**Table S2.** The effect of physicality (physical\emotional) as well as situation intensity and valence measured by dichotomous situation classification, and touch type on touch preference. Note that Study 3 did not distinguish situation valence dichotomously and did not distinguish situation physicality, and that Study 4 did not distinguish situation valence.

|  |  | b(SE) | 95% CI | t(df) | p | f2 |
| --- | --- | --- | --- | --- | --- | --- |
| Study 1 | Intercept | 3.759(.056) | 3.65,3.87 | 66.688(1470) | < .001*** | 0 |
|  | Intensity | 0.019(.036) | -0.05,0.09 | 0.528(1470) | .598 | 0 |
|  | Touch Type | 0.228(.035) | 0.16,0.30 | 6.539(1470) | < .001*** | .017 |
|  | Valence | -0.247(.035) | -0.32,-0.18 | -7.103(1470) | < .001*** | .02 |
|  | Physicality | -0.052(.035) | -0.12,0.02 | -1.485(1470) | .138 | .001 |
|  | Intensity*Touch Type | 0.262(.07) | 0.13,0.40 | 3.757(1470) | < .001*** | .006 |
|  | Intensity*Valence | -0.149(.072) | -0.29,-0.01 | -2.084(1470) | .037* | .002 |
|  | Touch Type*Valence | 0.117(.07) | -0.02,0.25 | 1.681(1470) | .093^†^ | .001 |
|  | Intensity*Physicality | -0.183(.072) | -0.32,-0.04 | -2.557(1470) | .011* | .005 |
|  | Touch Type*Physicality | -0.21(.07) | -0.35,-0.07 | -3.019(1470) | .003** | .004 |
|  | Valence*Physicality | -0.159(.07) | -0.30,-0.02 | -2.283(1470) | .023* | .002 |
|  | Intensity*Touch Type*Valence | 0.069(.139) | -0.20,0.34 | 0.494(1470) | .621 | 0 |
|  | Intensity*Touch Type*Physicality | 0.012(.139) | -0.26,0.29 | 0.087(1470) | .931 | 0 |
|  | Intensity*Valence*Physicality | -0.377(.143) | -0.66,-0.10 | -2.627(1470) | .009** | .006 |
|  | Touch Type*Valence*Physicality | -0.047(.139) | -0.32,0.23 | -0.336(1470) | .737 | 0 |
|  | Intensity*Touch Type*Valence*Physicality | -0.481(.279) | -1.03,0.07 | -1.725(1470) | .085^†^ | .001 |
| Study 2 touch provision | Intercept | 3.886(.066) | 3.76,4.02 | 58.616(2308) | < .001*** | 0 |
|  | Intensity | 0.004(.031) | -0.06,0.06 | 0.119(2308) | .905 | 0 |
|  | Touch Type | 0.389(.031) | 0.33,0.45 | 12.49(2308) | < .001*** | .036 |
|  | Valence | -0.144(.031) | -0.20,-0.08 | -4.614(2308) | < .001*** | .005 |
|  | Physicality | -0.106(.031) | -0.17,-0.05 | -3.421(2308) | .001*** | .003 |
|  | Intensity*Touch Type | 0.205(.062) | 0.08,0.33 | 3.302(2308) | .001*** | .003 |
|  | Intensity*Valence | -0.156(.062) | -0.28,-0.03 | -2.506(2308) | .012* | .001 |
|  | Touch Type*Valence | -0.094(.062) | -0.22,0.03 | -1.512(2308) | .131 | .001 |
|  | Intensity*Physicality | -0.22(.062) | -0.34,-0.10 | -3.54(2308) | < .001*** | .003 |
|  | Touch Type*Physicality | -0.109(.062) | -0.23,0.01 | -1.75(2308) | .080^†^ | .001 |
|  | Valence*Physicality | -0.109(.062) | -0.23,0.01 | -1.75(2308) | .080^†^ | .001 |
|  | Intensity*Touch Type*Valence | 0.045(.124) | -0.20,0.29 | 0.358(2308) | .720 | 0 |
|  | Intensity*Touch Type*Physicality | -0.064(.124) | -0.31,0.18 | -0.517(2308) | .605 | 0 |
|  | Intensity*Valence*Physicality | -0.322(.124) | -0.57,-0.08 | -2.586(2308) | .010** | .002 |
|  | Touch Type*Valence*Physicality | -0.01(.124) | -0.25,0.23 | -0.08(2308) | .937 | 0 |
|  | Intensity*Touch Type*Valence*Physicality | 3.886(.066) | 3.76,4.02 | 58.616(2308) | < .001*** | 0 |
| Study 2 touch reception | Intercept | 3.885(.067) | 3.75,4.02 | 58.133(2308) | < .001*** | 0 |
|  | Intensity | 0.025(.032) | -0.04,0.09 | 0.763(2308) | .446 | 0 |
|  | Touch Type | 0.495(.032) | 0.43,0.56 | 15.255(2308) | < .001*** | .055 |
|  | Valence | -0.202(.032) | -0.27,-0.14 | -6.216(2308) | < .001*** | .009 |
|  | Physicality | -0.002(.032) | -0.07,0.06 | -0.076(2308) | .939 | 0 |
|  | Intensity*Touch Type | 0.134(.065) | 0.01,0.26 | 2.059(2308) | .040* | .001 |
|  | Intensity*Valence | -0.186(.065) | -0.31,-0.06 | -2.86(2308) | .004** | .002 |
|  | Touch Type*Valence | -0.057(.065) | -0.18,0.07 | -0.877(2308) | .381 | 0 |
|  | Intensity*Physicality | -0.04(.065) | -0.17,0.09 | -0.61(2308) | .542 | 0 |
|  | Touch Type*Physicality | -0.158(.065) | -0.29,-0.03 | -2.441(2308) | .015* | .001 |
|  | Valence*Physicality | -0.166(.065) | -0.29,-0.04 | -2.555(2308) | .011* | .002 |
|  | Intensity*Touch Type*Valence | 0.064(.13) | -0.19,0.32 | 0.496(2308) | .620 | 0 |
|  | Intensity*Touch Type*Physicality | 0.089(.13) | -0.17,0.34 | 0.686(2308) | .492 | 0 |
|  | Intensity*Valence*Physicality | -0.173(.13) | -0.43,0.08 | -1.335(2308) | .182 | 0 |
|  | Touch Type*Valence*Physicality | 0.045(.13) | -0.21,0.30 | 0.343(2308) | .731 | 0 |
|  | Intensity*Touch Type*Valence*Physicality | -0.267(.26) | -0.78,0.24 | -1.03(2308) | .303 | 0 |
| Study 4 | Intercept | 53.316(4.608) | 44.22,62.41 | 11.571(181) | < .001*** | .002 |
|  | Intensity | -1.418(3.568) | -8.46,5.62 | -0.397(181) | .692 | 0 |
|  | Touch Type | 21.487(3.463) | 14.65,28.32 | 6.204(181) | < .001*** | .09 |
|  | Physicality | -13.255(3.59) | -20.34,-6.17 | -3.692(181) | < .001*** | .035 |
|  | Intensity*Touch Type | 9.438(6.927) | -4.23,23.11 | 1.362(181) | .175 | .004 |
|  | Intensity*Physicality | -0.362(7.137) | -14.44,13.72 | -0.051(181) | .960 | 0 |
|  | Touch Type*Physicality | 5.96(6.927) | -7.71,19.63 | 0.86(181) | .391 | .002 |
|  | Intensity*Touch Type*Physicality | -2.523(13.854) | -29.86,24.81 | -0.182(181) | .856 | 0 |

Note – High valence values refer to situations being perceived as positive.

**Injection phobia levels and cultural differences**

We note here the methods and results examining two exploratory hypotheses.

Hypotheses

1. Injection phobia (Study 3 exploratory hypothesis) – Injection phobia levels will moderate touch preference in Study 3. (Exploratory)
2. Culture (Study 4 exploratory hypothesis) - Culture will not moderate touch preference in Study 4.

Results

Injection phobia (Study 3 exploratory hypothesis)

Our hypothesis that injection phobia would moderate touch type and intensity effects was partly confirmed. Injection phobia levels did not moderate the effect of intensity on touch preference. They were associated with a weaker preference for handholding over stroking regardless of intensity, whether intensity was measured using ratings (b = -.009(SE = .003), t(402) = -3.007, p = .003) or dichotomously (b = -.01(SE = .003), t(402) = -3.242, p = .001). However, simple slope analyses revealed that the preference for handholding remained significant even when injection phobia was 2 standard deviations above the mean (b = .479(SE = .203), t(402) = 2.354, p = .019). Full tables are provided in Table S3.

Culture (Study 4 exploratory hypothesis)

Our hypothesis that culture would not moderate touch preference was confirmed. Culture, coded dichotomously as 0.5 for Arabic speakers and -0.5 for Hebrew speakers, was not associated with a preference for a specific type of touch, and did not moderate intensity effects. Culture was associated with higher ratings for touch in general, regardless of type, whether intensity was measured using ratings (b = 24.887(SE = 8.623), t(34) = 2.886, p = .007) or dichotomously (b = 25.416(SE = 8.641), t(34) = 2.941, p = .006). Full tables are provided in Table S4.

**Table S3.** Moderation by Injection phobia levels of the effect of touch type and intensity (measured by ratings or by dichotomous situation classifications) on subjective preference.

|  |  | b(SE) | 95% CI | t(df) | p | f^2^ |
| --- | --- | --- | --- | --- | --- | --- |
| Study 3 Intensity Rating | Intercept | 3.603(.111) | 3.38,3.82 | 32.399(402) | < .001*** | 0 |
|  | Intensity | 0.192(.051) | 0.09,0.29 | 3.751(402) | < .001*** | .019 |
|  | Touch Type | 1.069(.091) | 0.89,1.25 | 11.761(402) | < .001*** | .19 |
|  | Injection Phobia | 0.006(.004) | 0.00,0.01 | 1.656(49) | .104 | .024 |
|  | Intensity*Touch Type | 0.249(.102) | 0.05,0.45 | 2.435(402) | .015* | .008 |
|  | Intensity*Injection Phobia | 0.002(.002) | 0.00,0.01 | 0.961(402) | .337 | .001 |
|  | Touch Type*Injection Phobia | -0.01(.003) | -0.02,0.00 | -3.242(402) | .001** | .014 |
|  | Intensity*Touch Type*Injection Phobia | -0.001(.004) | -0.01,0.01 | -0.205(402) | .838 | 0 |
| Study 3 Dichotomous Intensity | Intercept | 3.52(.112) | 3.30,3.74 | 31.341(402) | < .001*** | 0 |
|  | Intensity | 0.5(.095) | 0.31,0.69 | 5.257(402) | < .001*** | .038 |
|  | Touch Type | 0.99(.095) | 0.80,1.18 | 10.412(402) | < .001*** | .148 |
|  | Injection Phobia | 0.006(.004) | 0.00,0.01 | 1.636(49) | .108 | .024 |
|  | Intensity*Touch Type | 0.471(.19) | 0.10,0.84 | 2.474(402) | .014* | .008 |
|  | Intensity*Injection Phobia | 0(.003) | -0.01,0.01 | 0.028(402) | .978 | 0 |
|  | Touch Type*Injection Phobia | -0.009(.003) | -0.02,0.00 | -3.007(402) | .003** | .012 |
|  | Intensity*Touch Type*Injection Phobia | -0.002(.006) | -0.01,0.01 | -0.271(402) | .787 | 0 |

**Table S4.** Moderation by culture of the effect of touch type and intensity (measured by ratings or by by dichotomous situation classifications) on subjective preference. (Culture coded 0.5 for Arabic speakers and -0.5 for Hebrew speakers).

|  |  | b(SE) | 95% CI | t(df) | p | f^2^ |
| --- | --- | --- | --- | --- | --- | --- |
| Study 4 Intensity Rating | Intercept | 52.19(4.311) | 43.68,60.70 | 12.105(182) | < .001*** | .006 |
|  | Intensity | 3.339(2.392) | -1.38,8.06 | 1.395(182) | .165 | .002 |
|  | Touch Type | 22.605(3.478) | 15.74,29.47 | 6.5(182) | < .001*** | .108 |
|  | Culture | 24.887(8.623) | 7.36,42.41 | 2.886(34) | .007** | .095 |
|  | Intensity*Touch Type | 0.864(4.716) | -8.44,10.17 | 0.183(182) | .855 | 0 |
|  | Intensity*Culture | 0.048(4.785) | -9.39,9.49 | 0.01(182) | .992 | 0 |
|  | Touch Type*Culture | 3.189(6.956) | -10.54,16.91 | 0.458(182) | .647 | .001 |
|  | Intensity*Touch Type*Culture | -2.61(9.431) | -21.22,16.00 | -0.277(182) | .782 | 0 |
| Study 4 Dichotomous Intensity | Intercept | 52.29(4.321) | 43.77,60.82 | 12.103(182) | < .001*** | .005 |
|  | Intensity | 0.05(3.587) | -7.03,7.13 | 0.014(182) | .989 | < 0 |
|  | Touch Type | 22.031(3.495) | 15.13,28.93 | 6.303(182) | < .001*** | .102 |
|  | Culture | 25.416(8.641) | 7.86,42.98 | 2.941(34) | .006** | .098 |
|  | Intensity*Touch Type | 9.02(6.991) | -4.77,22.81 | 1.29(182) | .199 | .004 |
|  | Intensity*Culture | -9.082(7.173) | -23.24,5.07 | -1.266(182) | .207 | .002 |
|  | Touch Type*Culture | 3.277(6.991) | -10.52,17.07 | 0.469(182) | .640 | .001 |
|  | Intensity*Touch Type*Culture | -1.362(13.982) | -28.95,26.23 | -0.097(182) | .923 | 0 |

**Situations**

Below are listed the situations provided in the study (Table S5), as well as their mean intensity and valence ratings (Table S6). Spearman correlations between mean ratings of situations in each study showed that the situations were ranked extremely consistently between the studies (.985 for intensity and .994 for valence).

**Table S5**

|  | Physical Negative | Emotional Negative | Physical Positive | Emotional Positive |
| --- | --- | --- | --- | --- |
| Intense | A: You are at the dentist's clinic. Imagine that you have just undergone a painful dental procedure. (1)  B: You are at home. Imagine that you have just fell down some stairs and landed on your knees. (2) | A: You are at home. Imagine that you are about to give an online presentation to a large audience. You are not sure you have prepared enough and are very anxious. (5)  B: You are at home. Imagine that you have just received news that you have failed an important test. (6) | A: You are at a dance party. Imagine that one of your favorite songs has come up. It's a fast and exciting song and you are dancing intensely. (9)  B: You are at home. Imagine that you're eating food you brought from a great restaurant and it tastes amazing.* (10) | A: You are at home. Imagine that you were just notified that you've won 300$ on a bet. (13)  B: You are climbing a hill on a short trip. Imagine that you are seeing a beautiful landscape on the top of the hill.* (14) |
| Non-Intense | A: You are at home. Imagine that you have caught a cold and are not feeling well. (3)  B: You've eaten something bad and are resting at home. There are no severe symptoms, but you generally feel a bit nauseous and tired. (4) | A: You are at home. Imagine that you have just been informed that you have lost 20$ on a bet. (7)  B: You are at home. Imagine that a person close to you is about to move to a faraway country for a long time. (8) | A: You are at home. Imagine that you've just finished getting dressed after taking a nice shower and you're feeling refreshed. (11)  B: Imagine that you've just woken up on a weekend morning after having a great night's sleep.* (12) | A: You are at home. Imagine that you have just gotten home after a great day at work / school. (15)  B: You are at home. Imagine that you have just read a heartwarming article about someone who overcame a harsh disease and went on to do amazing things.* (16) |

* These situations’ intensity was originally coded differently for Study 1 (intense situations were originally coded as non-intense and vice versa).

**Table S6**

| Situation # | Classification | Intensity | | Valence | |
| --- | --- | --- | --- | --- | --- |
|  |  | Study 1 mean(SD) | Study 2 mean(SD) | Study 1 mean(SD) | Study 2 mean(SD) |
| 1 | Physical, negative, intense | 3.23(.67) | 3.22(.81) | 1.89(1.08) | 2.07(1.4) |
| 2 | Physical, negative, intense | 3.09(.73) | 3.15(.75) | 1.71(.87) | 1.83(1.07) |
| 3 | Physical, negative, non-intense | 2.11(.68) | 2.11(.72) | 2.3(.67) | 2.72(1.23) |
| 4 | Physical, negative, non-intense | 2.21(.56) | 2.2(.75) | 2.23(.71) | 2.6(1.05) |
| 5 | Emotional, negative, intense | 3.06(.7) | 3.1(.76) | 2.53(1.12) | 2.74(1.35) |
| 6 | Emotional, negative, intense | 3(.67) | 3.07(.73) | 1.64(.65) | 1.91(1.25) |
| 7 | Emotional, negative, non-intense | 1.61(.64) | 1.66(.68) | 2.78(.68) | 3.09(.91) |
| 8 | Emotional, negative, non-intense | 2.94(.83) | 2.99(.71) | 2.12(.92) | 2.36(1.37) |
| 9 | Physical, positive, intense | 2.72(.73) | 2.64(.83) | 6.05(1.02) | 5.94(.84) |
| 10 | Physical, positive, intense | 2.22(.91) | 2.23(.87) | 6.15(.8) | 6.09(.86) |
| 11 | Physical, positive, non-intense | 1.52(.76) | 1.68(.88) | 5.81(.81) | 5.57(.85) |
| 12 | Physical, positive, non-intense | 1.48(.85) | 1.71(.92) | 6.05(.91) | 5.88(.99) |
| 13 | Emotional, positive, intense | 2.81(.73) | 2.86(.74) | 6.5(.76) | 6.21(1.18) |
| 14 | Emotional, positive, intense | 2.54(.79) | 2.51(.83) | 6.31(.77) | 6.12(.94) |
| 15 | Emotional, positive, non-intense | 1.84(.76) | 2(.869) | 5.97(.8) | 5.89(.84) |
| 16 | Emotional, positive, non-intense | 2.07(.84) | 2.02(.821) | 5.85(1.03) | 5.79(.95) |

**Figure S1**


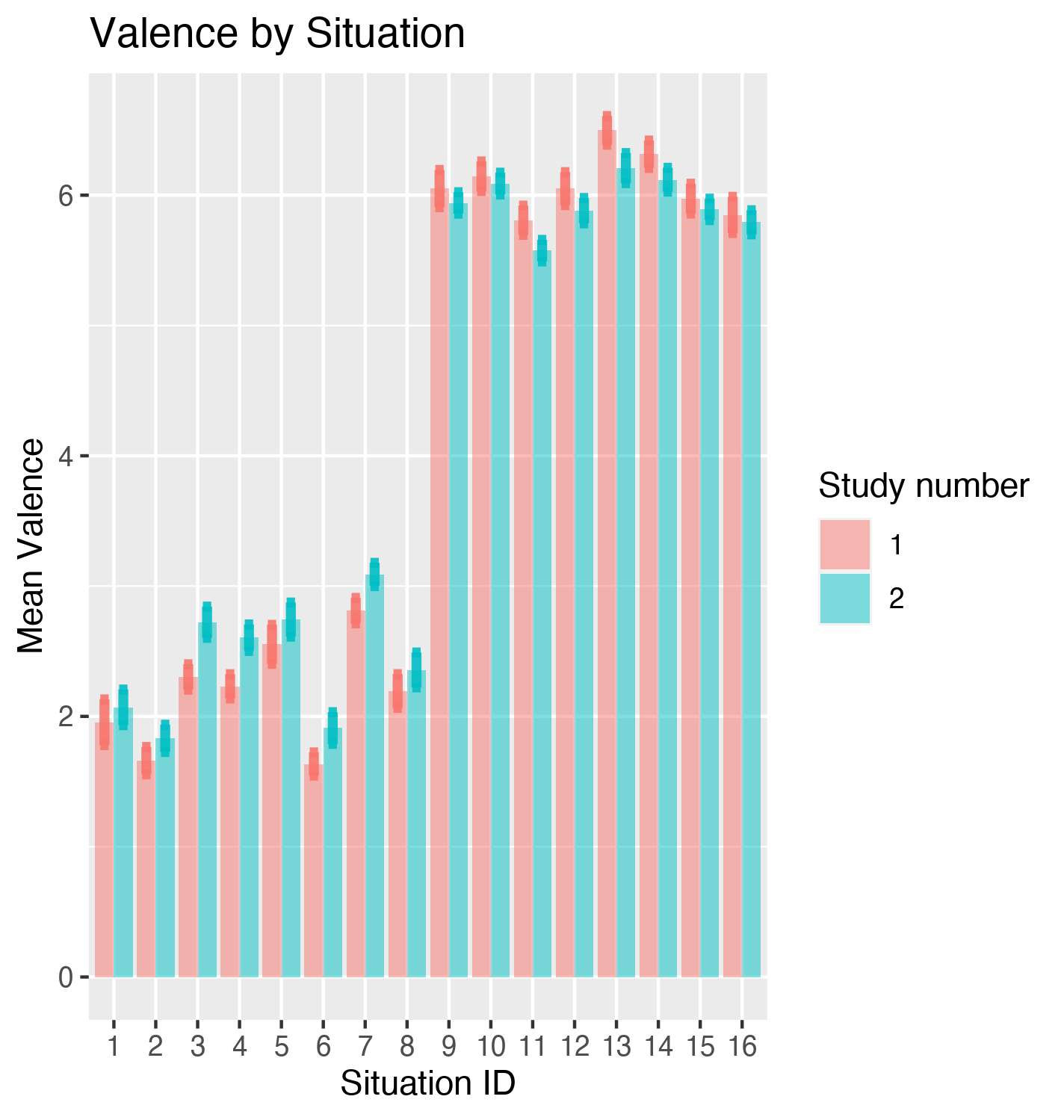

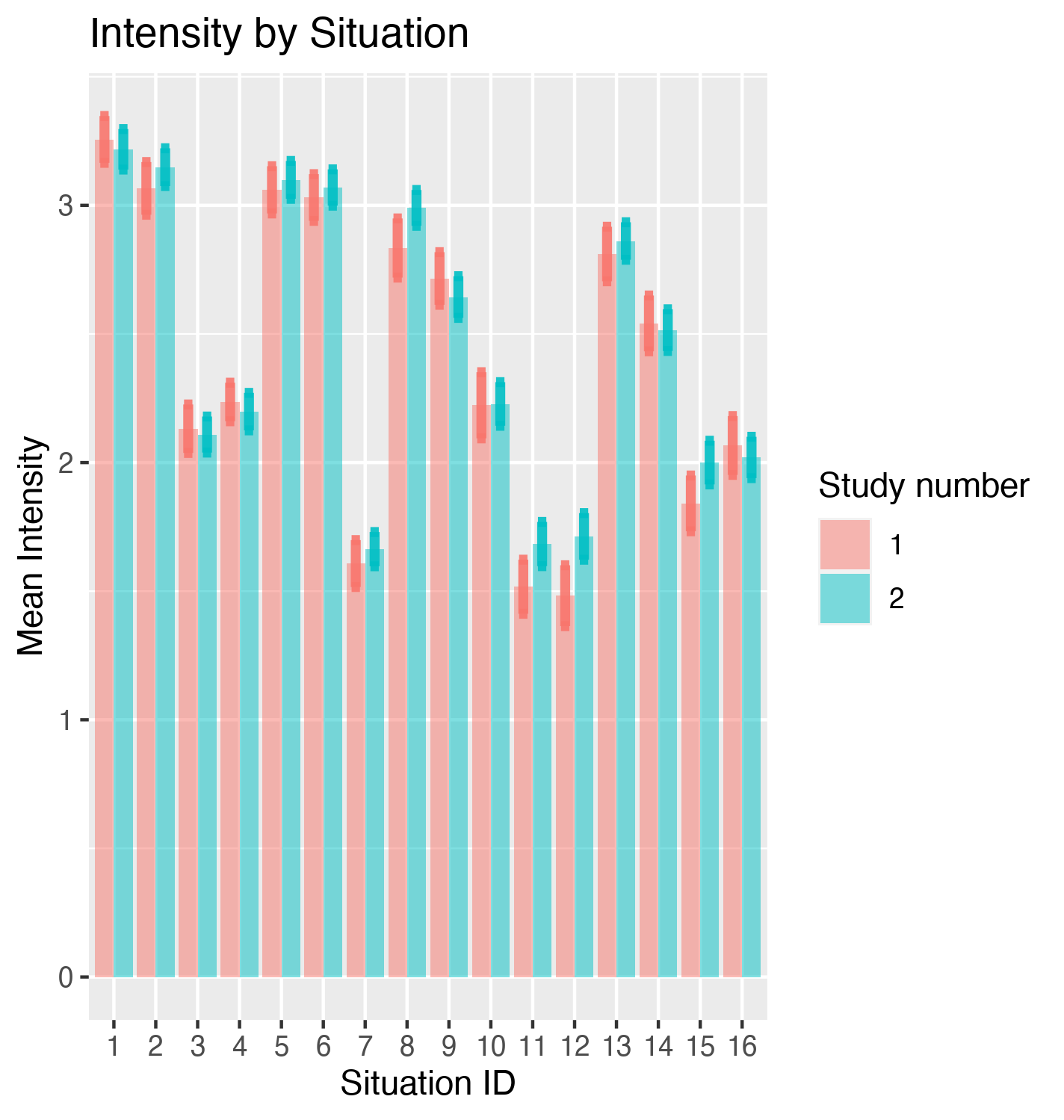


**Table S7**

Significance of effects across studies

|  | Study 1 part 1 (forced choice) | Study 1 part 2 (rating) | Study 2 part 1 (reception) | Study 2 part 2 (provision) | Study 3 | Study 4 part 1 (recall) | Study 4 part 2 (rating) |
| --- | --- | --- | --- | --- | --- | --- | --- |
| Handholding Preferred | Sig. | Sig. | Sig. | Sig. | Sig. | Sig. | Sig. |
| Handholding preferred more in intense situations | Sig. | Sig. | Sig. | Sig. | Sig. | N/A | n.s. |
| All touch rated higher in negative situations | N/A | Sig. | Sig. | n.s. (Significant with dichotomous measures) | n.s. | N/A | N/A |
| All touch rated higher in emotional situations | N/A | n.s. | n.s. | Sig. | N/A | N/A | Sig. |
| All touch rated higher in negative physical situations | N/A | Sig. | Sig. | Sig. only with continuous measures | N/A | N/A | N/A |
| Handholding preferred more in positive situations | N/A | Sig. | n.s. | n.s. | n.s. | N/A | N/A |
| Handholding preferred more in emotional situations | N/A | Sig. | Sig. | Sig. (not significant with dichotomous measures) | N/A | N/A | n.s. |
| Intensity, touch type and valence three-way interaction | N/A | n.s. | n.s. | Sig. (not significant with dichotomous measures) | n.s. | N/A | N/A |
| Intensity, touch type and physicality three-way interaction | N/A | n.s. | n.s. | n.s. (significant with dichotomous measures) | N/A | N/A | n.s. |

**Robustness Tests**

Below are results for repeat analyses of the main two hypotheses (handholding preferred over stroking, with an effect that increases with intensity). Tables S8 and S9 test the main analyses with saturated random slopes (i.e., allowing for a random slope for each effect. Note that some of effect sizes are missing as some of the secondary models required for our calculations of effect sizes (i.e., models omitting one of the variables) did not converge.

Simple repeated ANOVA analyses of the main hypotheses using discrete situation intensity ratings revealed the same results as all other analyses. Ratings for handholding were significantly higher than for stroking in all studies (Study 1 F(1,97) = 23.286, p < .001; Study 2 provision F(1,100) = 41.27, p < .001; Study 2 reception F(1,100) = 63.97, p < .001; Study 3 F(1,50) = 43.06, p < .001; Study 4 F(1,34) = 20.7, p < .001). The interaction between touch type and intensity was significant in all studies except Study 4 (Study 1 F(1,98) = 32.9, p < .001; Study 2 provision F(1,100) = 14.74, p < .001; Study 2 reception F(1,100) = 8.329, p = .005, Study 3 F(1,50) = 8.578, p = .005, Study 4 F(1,28) = 2.13, p = .156).

As the fully saturated cumulative link model for the second part of Study 2 did not converge when using participant-rated intensity, we performed additional robustness checks. First, we ran an intercept-only ordinal model which converged and demonstrated identical significant effects to all other analyses. We also determined that the convergence issues were due to data from a single participant (participant 952408). Running the fully saturated model without this participant converged with identical significant effects to all other analyses. To ensure that the effects were not artifacts caused by removing this participant’s data, we also ran a fully saturated model with this participant while artificially increasing one of this participants’ ratings for stroking in one of the intense situations from 1 to 3 (i.e. weakening both of the significant effects, which state that ratings for stroking are lower than for handholding, especially in intense situations); This analysis also converged with identical significant effects to all other analyses.

**Table S8.** The effect of intensity measured by intensity ratings and touch type on touch preference analyzed using saturated random slopes.

|  |  | b(SE) | 95% CI | t(df) | p | f^2^ |
| --- | --- | --- | --- | --- | --- | --- |
| Study 1 | Intercept | 3.759(.057) | 3.65,3.87 | 66.23(1482) | < .001*** |  |
|  | Intensity | 0.12(.028) | 0.06,0.18 | 4.236(1482) | < .001*** |  |
|  | Touch Type | 0.226(.047) | 0.13,0.32 | 4.822(1482) | < .001*** |  |
|  | Intensity*Touch Type | 0.131(.044) | 0.04,0.22 | 2.977(1482) | .003** |  |
| Study 2 touch provision | Intercept | 4.01(.073) | 3.87,4.15 | 54.779(2320) | < .001*** |  |
|  | Intensity | 0.138(.035) | 0.07,0.21 | 3.981(2320) | < .001*** |  |
|  | Touch Type | 0.483(.075) | 0.34,0.63 | 6.424(2320) | < .001*** |  |
|  | Intensity*Touch Type | 0.158(.044) | 0.07,0.24 | 3.585(2320) | < .001*** | .004 |
| Study 2 touch reception | Intercept | 3.885(.062) | 3.76,4.01 | 62.789(2320) | < .001*** |  |
|  | Intensity | 0.055(.029) | 0.00,0.11 | 1.869(2320) | .062^†^ |  |
|  | Touch Type | 0.495(.062) | 0.37,0.62 | 7.996(2320) | < .001*** |  |
|  | Intensity*Touch Type | 0.108(.037) | 0.04,0.18 | 2.915(2320) | .004** |  |
| Study 3 | Intercept | 3.603(.104) | 3.40,3.81 | 34.637(405) | < .001*** |  |
|  | Intensity | 0.239(.067) | 0.11,0.37 | 3.551(405) | < .001*** |  |
|  | Touch Type | 1.069(.163) | 0.75,1.39 | 6.562(405) | < .001*** |  |
|  | Intensity*Touch Type | 0.24(.079) | 0.08,0.39 | 3.037(405) | .003** |  |
| Study 4 | Intercept | 50.906(4.677) | 41.68,60.13 | 10.884(185) | < .001*** | .002 |
|  | Intensity | 3.569(2.392) | -1.15,8.29 | 1.492(185) | .137 |  |
|  | Touch Type | 24.271(5.061) | 14.29,34.25 | 4.796(185) | < .001*** |  |
|  | Intensity*Touch Type | 0.184(3.986) | -7.68,8.05 | 0.046(185) | .963 | 0 |
| Study 1 with omitted participants | Intercept | 3.743(.057) | 3.63,3.85 | 65.597(1512) | < .001*** |  |
|  | Intensity | 0.119(.028) | 0.06,0.17 | 4.29(1512) | < .001*** |  |
|  | Touch Type | 0.21(.048) | 0.12,0.30 | 4.421(1512) | < .001*** |  |
|  | Intensity*Touch Type | 0.148(.044) | 0.06,0.23 | 3.324(1512) | .001*** |  |

**Table S9.** The effect of intensity measured by dichotomous situation classifications and touch type on touch preference analyzed using saturated random slopes.

|  |  | b(SE) | 95% CI | t(df) | p | f^2^ |
| --- | --- | --- | --- | --- | --- | --- |
| Study 1 | Intercept | 3.761(.057) | 3.65,3.87 | 66.354(1482) | < .001*** |  |
|  | Intensity | 0.025(.039) | -0.05,0.10 | 0.63(1482) | .529 |  |
|  | Touch Type | 0.229(.047) | 0.14,0.32 | 4.87(1482) | < .001*** |  |
|  | Intensity*Touch Type | 0.272(.071) | 0.13,0.41 | 3.85(1482) | < .001*** |  |
| Study 2 touch provision | Intercept | 3.886(.063) | 3.76,4.01 | 62.004(2320) | < .001*** |  |
|  | Intensity | 0.004(.039) | -0.07,0.08 | 0.096(2320) | .924 |  |
|  | Touch Type | 0.389(.061) | 0.27,0.51 | 6.403(2320) | < .001*** |  |
|  | Intensity*Touch Type | 0.205(.059) | 0.09,0.32 | 3.465(2320) | .001*** |  |
| Study 2 touch reception | Intercept | 3.885(.062) | 3.76,4.01 | 62.79(2320) | < .001*** |  |
|  | Intensity | 0.025(.043) | -0.06,0.11 | 0.575(2320) | .566 |  |
|  | Touch Type | 0.495(.062) | 0.37,0.62 | 7.996(2320) | < .001*** |  |
|  | Intensity*Touch Type | 0.134(.061) | 0.01,0.25 | 2.184(2320) | .029* |  |
| Study 3 | Intercept | 3.52(.101) | 3.32,3.72 | 34.969(405) | < .001*** | .042 |
|  | Intensity | 0.5(.121) | 0.26,0.74 | 4.135(405) | < .001*** | .037 |
|  | Touch Type | 0.99(.162) | 0.67,1.31 | 6.125(405) | < .001*** | .159 |
|  | Intensity*Touch Type | 0.471(.161) | 0.15,0.79 | 2.929(405) | .004** | .008 |
| Study 4 | Intercept | 51.065(4.721) | 41.75,60.38 | 10.817(185) | < .001*** |  |
|  | Intensity | 0.379(3.201) | -5.94,6.69 | 0.119(185) | .906 |  |
|  | Touch Type | 23.54(5.127) | 13.43,33.65 | 4.592(185) | < .001*** |  |
|  | Intensity*Touch Type | 7.55(6.274) | -4.83,19.93 | 1.203(185) | .230 |  |
| Study 1 with omitted participants | Intercept | 3.744(.057) | 3.63,3.86 | 66.005(1512) | < .001*** |  |
|  | Intensity | 0.017(.039) | -0.06,0.09 | 0.443(1512) | .658 |  |
|  | Touch Type | 0.212(.048) | 0.12,0.31 | 4.409(1512) | < .001*** |  |
|  | Intensity*Touch Type | 0.279(.07) | 0.14,0.42 | 3.995(1512) | < .001*** |  |

**Simple Slopes**

**Table S10.** Simple slope analyses for the interaction between physicality and touch type in Studies 1 and 2

|  |  | b(SE) | t(df) | p |
| --- | --- | --- | --- | --- |
| Study 1 | Higher ratings for handholding (vs. stroking) in emotional situations | 0.33(0.05) | 6.35(1470) | < .001*** |
|  | Higher ratings for handholding (vs. stroking) in physical situations | 0.12(0.06) | 2.11(1470) | .035* |
|  | Higher ratings for stroking in physical (vs. emotional) situations | 0.08(0.05) | 1.44(1470) | .149 |
|  | Higher ratings for handholding in physical (vs. emotional) situations | -0.13(0.05) | -2.49(1470) | .013* |
| Study 2 touch provision | Higher ratings for handholding (vs. stroking) in emotional situations | 0.42(0.05) | 9.24(2308) | < .001*** |
|  | Higher ratings for handholding (vs. stroking) in physical situations | 0.28(0.05) | 5.91(2308) | < .001*** |
|  | Higher ratings for stroking in physical (vs. emotional) situations | -0.04(0.04) | -1.10(2308) | .269 |
|  | Higher ratings for handholding in physical (vs. emotional) situations | -0.18(0.05) | -3.37(2308) | .001*** |
| Study 2 touch reception | Higher ratings for handholding (vs. stroking) in emotional situations | 0.55(0.05) | 11.48(2308) | < .001*** |
|  | Higher ratings for handholding (vs. stroking) in physical situations | 0.41(0.05) | 8.24(2308) | < .001*** |
|  | Higher ratings for stroking in physical (vs. emotional) situations | 0.05(0.04) | 1.23(2308) | .218 |
|  | Higher ratings for handholding in physical (vs. emotional) situations | -0.09(0.06) | -1.62(2308) | .106 |

**Full Data Plots**

**Figure S2.** Study 1 touch ratings per participant by touch type and intensity


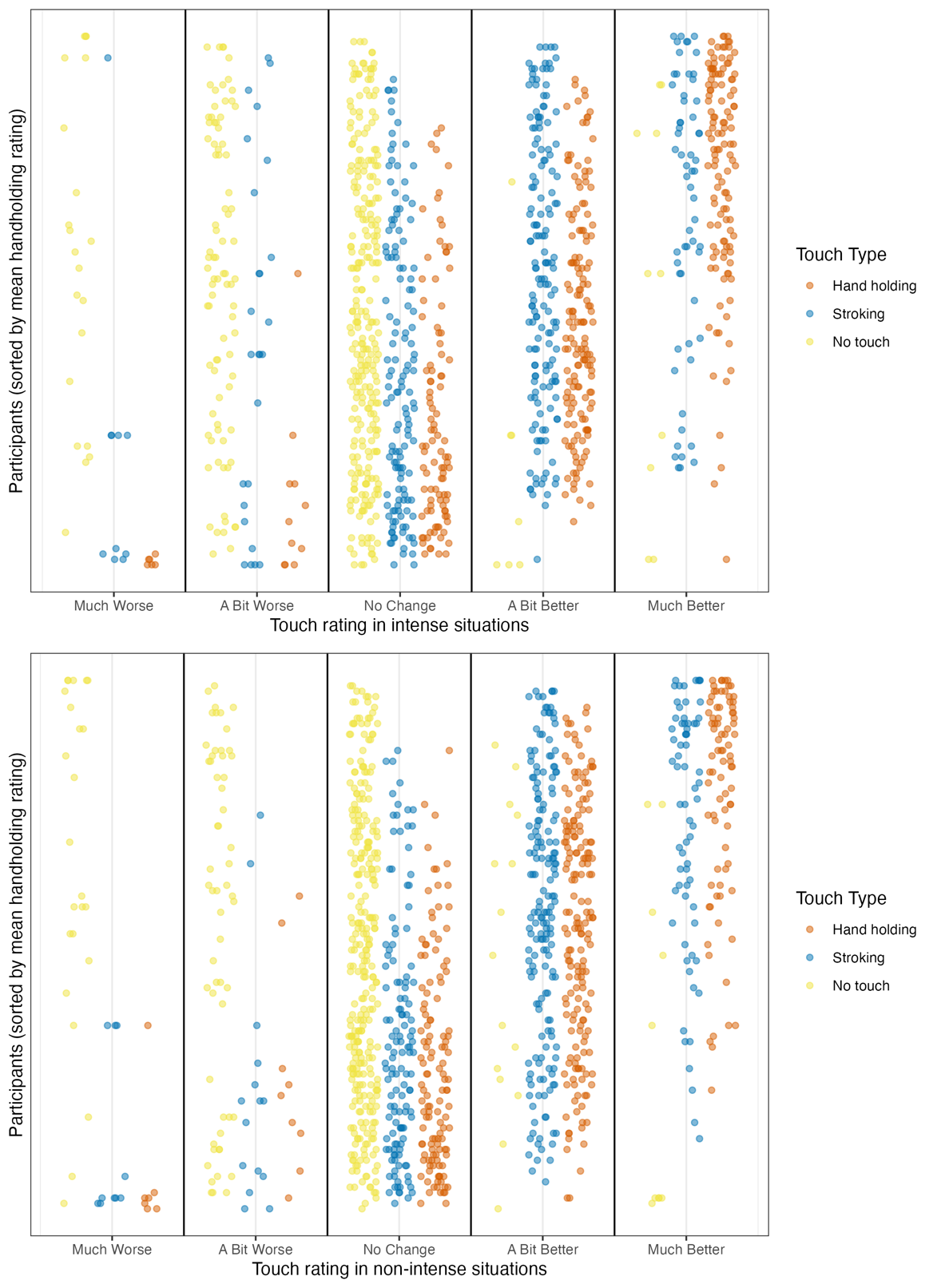


Participants are arranged along the y axis such that specific heights correspond to specific participants. Participants are sorted that participants with high mean ratings for handholding are at the top. Touch ratings indicate the way participants thought their feelings would change if they received that type of touch.

**Figure S3.** Study 2 touch reception ratings per participant by touch type and intensity


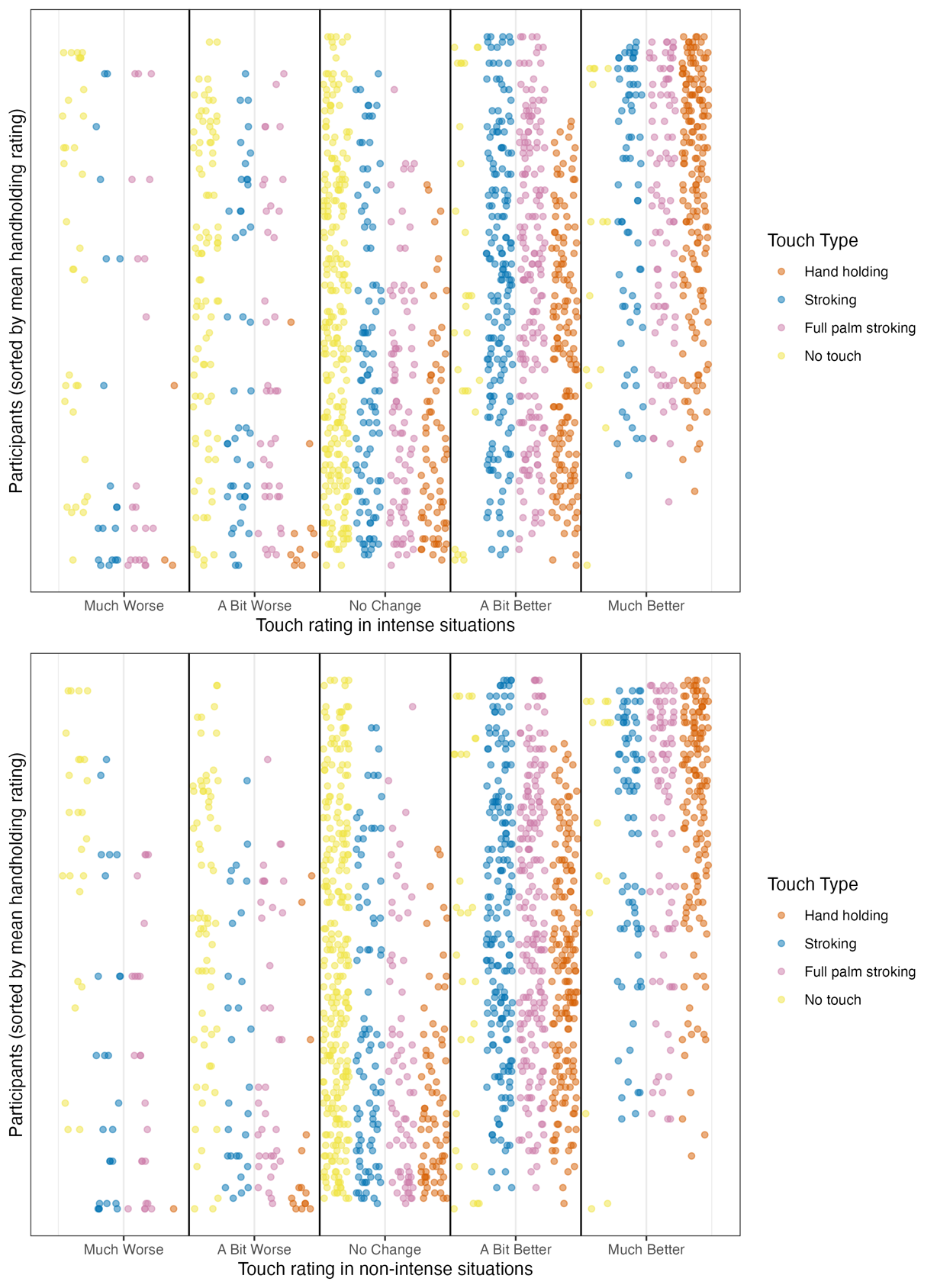


Participants are arranged along the y axis such that specific heights correspond to specific participants. Participants are sorted that participants with high mean ratings for handholding are at the top. Touch ratings indicate the way participants thought their feelings would change if they received that type of touch.

**Figure S4.** Study 2 touch provision ratings per participant by touch type and intensity


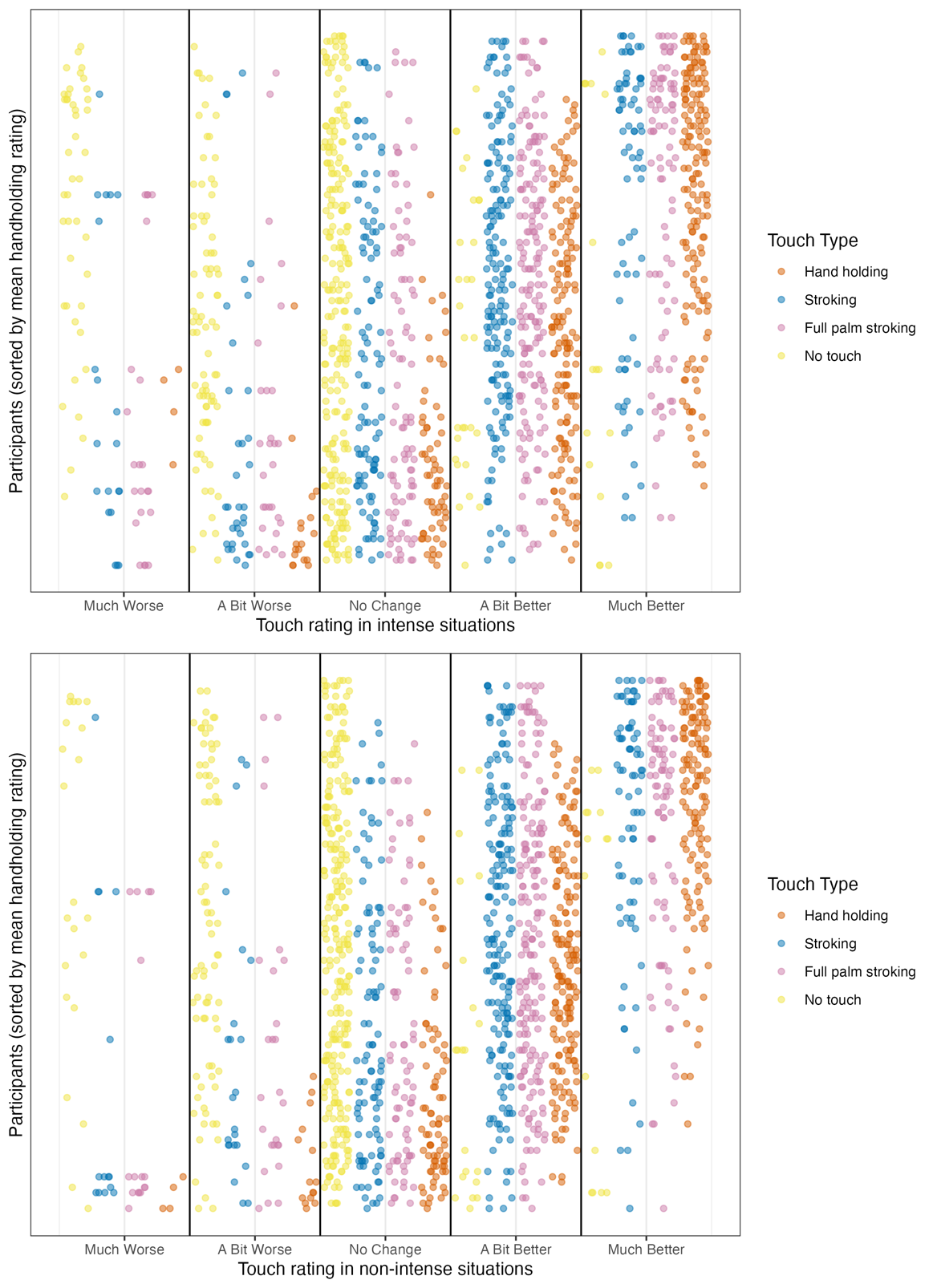


Participants are arranged along the y axis such that specific heights correspond to specific participants. Participants are sorted that participants with high mean ratings for handholding are at the top. Touch ratings indicate the way participants thought their partners’ feelings would change if they received that type of touch.

**Figure S5.** Study 3 touch ratings per participant by touch type and intensity


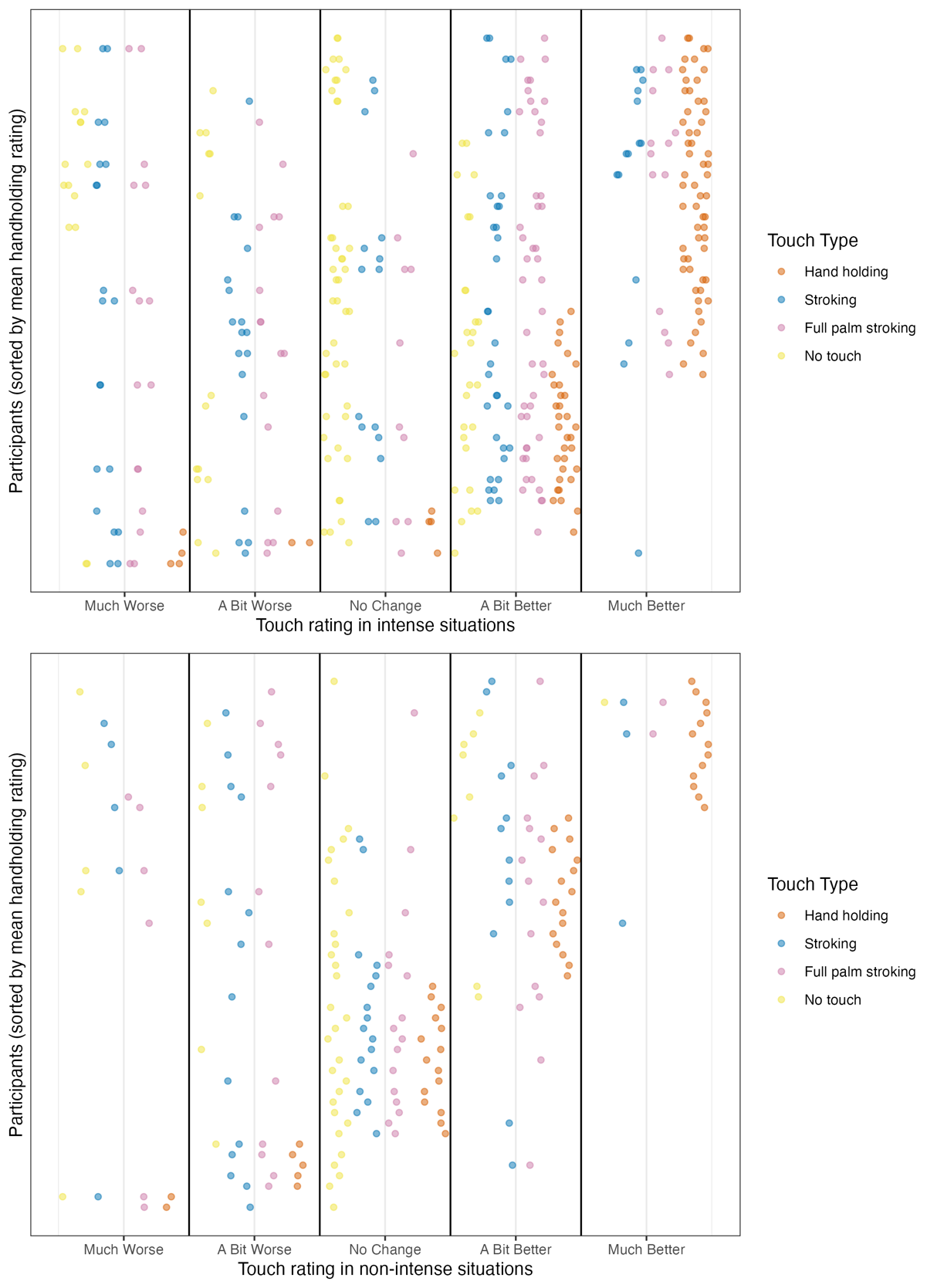


Participants are arranged along the y axis such that specific heights correspond to specific participants. Participants are sorted that participants with high mean ratings for handholding are at the top. Touch ratings indicate the way participants thought their feelings would change if they received that type of touch.

**Figure S6.** Study 4 touch ratings per participant by touch type and intensity


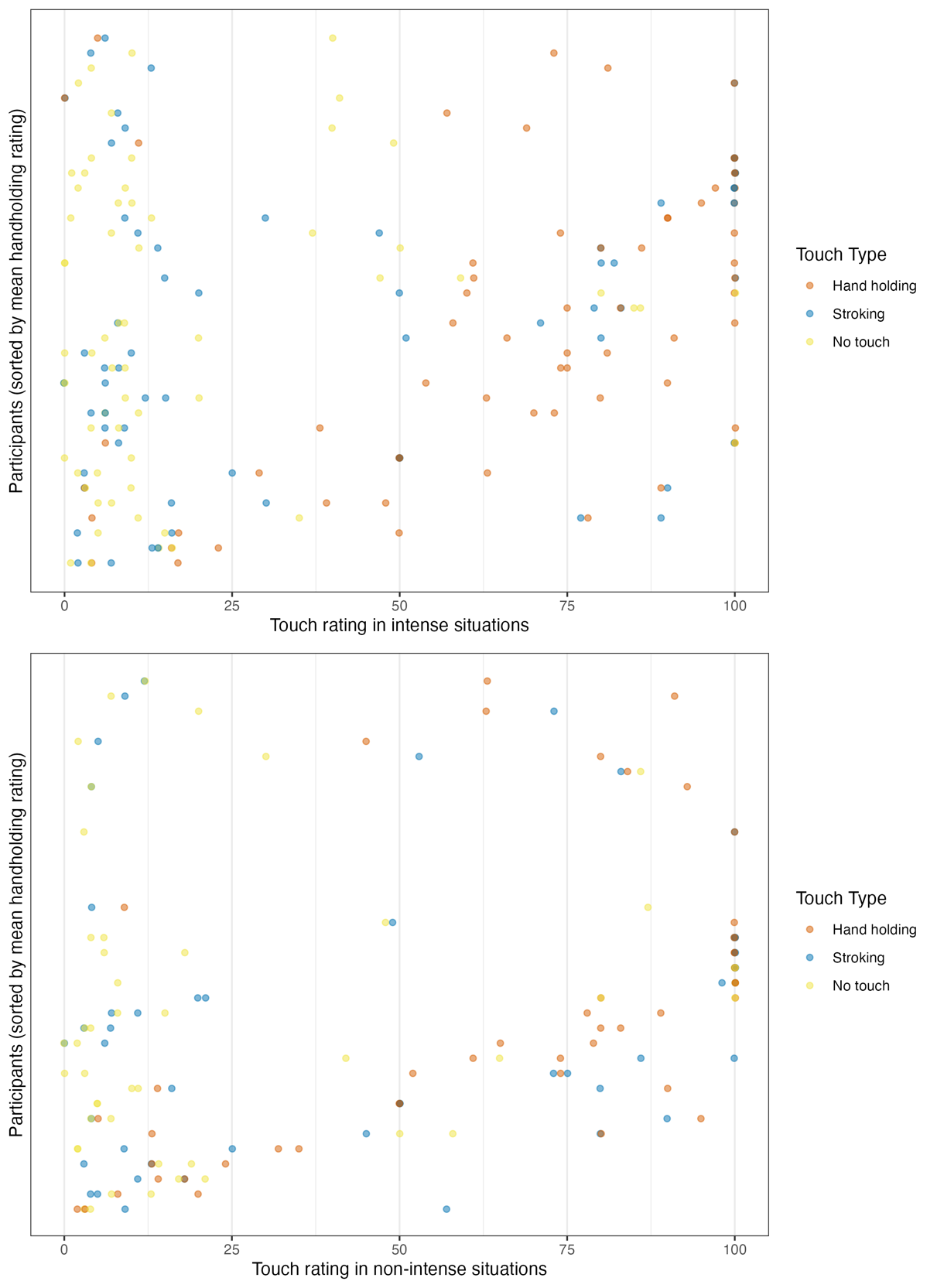


Participants are arranged along the y axis such that specific heights correspond to specific participants. Participants are sorted that participants with high mean ratings for handholding are at the top. Touch ratings indicate the way participants thought their feelings would change if they received that type of touch.
