# Supplementary material for: I wanna hold your hand: Handholding is preferred over gentle stroking for emotion regulation
Source: PLoS One. 2023 Apr 6;18(4):e0284161. doi: 10.1371/journal.pone.0284161 (PMC10079127; doi:10.1371/journal.pone.0284161)
Supplement: Attachment — Submitted filename: Consoling touch reviewer response final.docx [file pone.0284161.s002.docx]

We wish to thank the reviewer for their insightful comments. We read the comments carefully and incorporated the feedback into the manuscript. Below we detail our response to each point.

**The authors present a series of online studies where handholding is compared to arm stroking, in different intensity situations. They make the excellent distinction that handholding is a truly two-way (active/giving and receiving touch) interaction, whereas stroking is much more one-way (passive/receiving touch). The authors could make even more of this point throughout, where touch becomes a stronger form of communication in handholding. I congratulate the authors on the open way their studies are presented, yet the organization of the results is confusing. Overall, I am not convinced about the statistical analyses used. They seem incomplete, yet repetitive, and do not seem to correct for multiple tests, as well as using unjustified one-way t-tests. Also, the grounding of the work in previous literature needs to be revisited. However, the manuscript is novel and opens up new ideas into social touch mechanisms, adding new insights to the literature.

Introduction
- I do not think the study is well-grounded in the actual findings on affective touch. Many studies investigate the pleasantness of stroking touch and a few have conducted microneurography and shown that C-tactile fibers fire well to slow, gentle touch. However, C-tactile afferents are just one part of affective touch. The authors say, ‘Gentle stroking entails slowly stroking skin regions with C-tactile afferents…’ but this is imprecise, as gentle stroking will readily activate all types of mechanoreceptive afferent. Although CTs fire well to gentle skin stroking, it is clear that affective touch is not strictly linked to CT firing, where the relationship breaks down when other factors are added. Cognitive factors can influence gentle stroking perception, e.g. changes in pleasantness with different odors (Croy et al, 2014 Plos One). Such a breakdown in the CT vs. affective touch relationship has also been demonstrated, when touch was delivered at different temperatures and CT firing no longer correlated with pleasantness (Ackerley et al, 2014 J Neurosci). Further, a number of studies have shown little differences in the perception of pleasant touch on hairy arm and glabrous hand skin (for an overview, see Cruciani et al, 2021 Neurosci Biobehav Rev). Thus, affective touch is more complicated than simply CT afferent firing.**

We agree that the original introduction might not have been clear enough on these details, and we thank the reviewer for their helpful suggestion as to clarifying them. First, we now emphasize that C-touch does not only involve CT-afferents:

*Gentle stroking entails slowly stroking skin regions with C-tactile afferents [9], such as hairy skin on the forearm. Although slow stroking activates all types of mechanoreceptive afferents, it is also referred to as C-touch as C fibers respond optimally to this type of touch. At normal skin temperature (but not at very high or low temperatures), CT afferent firing is correlated with touch pleasantness [10]. Other studies have found that this kind of stroking, activates regions in the posterior insular cortex and the mid-anterior orbitofrontal cortex more than other types of touch [11,12]. Moreover, stroking is uniquely associated with elevated oxytocin levels [13] and activates social reward mechanisms [14].*

We have also mentioned, when discussing studies comparing the types of touch, the null findings concerning pleasantness:

*Existing studies in the field have focused on testing the effectiveness of touch as a method of emotion regulation, with most studies comparing stroking of the forearm to stroking or touching of the hand, but not to handholding. Studies comparing general pleasantness of touch between touching arm and hand skin found little difference [18]. In one of the only studies directly comparing the emotion regulation properties of stroking of the forearm and handholding, Reddan and colleagues [17] compared participants who received painful thermal stimulation to their legs, while their romantic partners supported them via gentle stroking of the forearm (i.e., C-touch), handholding or no touch. When measuring pain subjectively and through skin conductance response, no significant differences were found between handholding and gentle stroking, although effects of handholding were greater in every case.*

Finally, we have reworded a statement in the discussion, instead of mentioning that stroking relies “mainly” on bottom-up processes we now merely state that in stroking bottom-up processes “may play a larger part” [than in handholding].

**- In the next sentence, they say ‘…activates regions in the posterior insular cortex and the mid-anterior orbitofrontal cortex that are not activated by other types of touch’. Again, this is imprecise, as it is well-known that posterior insula is activated by all types of touch and the meta-analysis by Morrison (2016, Hum Brain Map) found that in general, posterior insula is more likely to be activated for affective touch, S1 for discriminative touch, whereas S2 is well-engaged in both; however, in no way were areas fully selective for one type of touch or the other.**

We appreciate this comment, and have changed the wording of the sentence, as well as citing Morrison (2016): “Studies have found that this kind of stroking, also referred to as C-touch, activates regions in the posterior insular cortex and the mid-anterior orbitofrontal cortex more than other types of touch [10,11]”

**- The authors say ‘no significant differences were found between handholding and gentle stroking, although effects of handholding were greater in every case’ for the work by Reddan et al (2020); however, if there were no significant differences in this work, they cannot claim that the effects of handholding were greater.**

We have now removed the qualifier, the sentence simply states that “When measuring pain subjectively and through skin conductance response, no significant differences were found between handholding and gentle stroking.”

**- The paper by Schirmer et al (2021, Emotion) on different types of touch may be useful to add to the introduction, in terms of the topographies of touch with others and the social implications.**

We thank the reviewer for pointing us in the direction of this paper, and we have now included it in in the introduction.

**Methods
- Would it be possible to add age details (e.g. range) for each study? I am also presuming that the sample for Study 4 was only females, but it would be good to state this too.**

Unfortunately, due to privacy considerations we did not collect age data from participants in Studies 1-3. We do now report age details for Study 4, as well as explicitly stating that all participants were female.

**- It would be good to have the ethical section at the beginning of the methods, as normally done.**

We have moved the ethics section as requested.

**Results
- Please can the number of decimal places be reduced in the tables? Only one (maybe two) decimal places are relevant for the data, too many numbers make the table difficult to interpret and I do not think such accuracy is relevant for these ratings (also for the t values).**

We have made these adjustments in all tables (except for p values for which we have kept three decimal places)

**- I do not understand why the data have been analyzed twice. Two mixed lineal models were conducted with intensity as a dichotomous variable and as a continuous variable, but there is no clear reason for this and it confuses the results.**

We agree that this double analysis may be confusing. The continuous analysis is somewhat more precise, while the dichotomous analysis is more closely tied to the experimental manipulation (the choice of scenarios). To resolve this, since the results are very similar, we have added a more detailed explanation of the double analysis strategy, and have moved the dichotomous analyses to the supplemental material. The new explanation of the analysis strategy is as follows:

*We conducted two mixed linear model analyses for each study. In the first, intensity was a measured continuous variable – the intensity of the situation as self-reported by participants. In the second, intensity was the pre-assigned intensity of the situations. For brevity, we report here only the findings for continuous intensity; full results for dichotomous intensity are reported in the supplemental material. Almost all results were significant in both analyses or non-significant in both analyses, and we state whenever there was a difference in significance.*

**- On p.22, I do not understand why the authors say (two times) that ‘this was only true in a one-tailed significance test’ and have p values of >0.05. I do not think they are justified in using a one-tailed t-test (why not corrected, post-hoc tests) and these p values are not significant. Also, just underneath, a p value of 0.094 is quoted as significant, when it is not.**

We agree, and do not treat these results as significant any more. The paragraph now reads as follows:

*Simple slope analyses on the interaction effects found in Studies 1-3 revealed that in almost all studies, handholding was rated as significantly higher than stroking regardless of intensity (the sole exception was that when analyzing Study 1 using dichotomous intensity classification, for non-intense situations, the difference was in the same direction but not significant, p = .059). Ratings for handholding almost always increased significantly with intensity (the sole exception was that when analyzing Study 2 using dichotomous intensity classification, only for touch reception, the effect was in the same direction but not significant, p = .088). Ratings for stroking never significantly increased with intensity when intensity was rated by participants. When intensity was measured using situation classifications results were unclear – ratings for stroking significantly decreased with intensity in Study 1 and when examining touch provision in Study 2; no effect was found when examining touch reception in Study 2; and ratings significantly increased with intensity in Study 3.*

**- Top of p.23, why are the results for non-intense situations not stated?**

We appreciate the reviewer alerting us to this error. We now have included chi-square analyses for a main effect, an interaction effect, and simple effects. The paragraph now reads:

*In Study 1, beyond asking participants to rate each touch type, we also asked them to choose a single type of touch they would want to receive the most. Out of 395 situations classified as intense, handholding was preferred 216 times (54.7%), stroking 87 times (22%), and no touch 92 times (23.3%). Out of 389 situations classified as non-intense, handholding was preferred 133 times (34.2%), stroking 164 times (42.2%) and no touch 92 times (23.7%). The results are presented in Figure 3. Chi-square tests for independence confirmed that handholding was preferred over stroking overall (χ2(1) = 16.007, p < .001) and that there was an interaction effect between touch type and intensity (χ2(2) = 43.317, p < .001)*^[1]^*. Follow-up tests for simple effects found that in intense situations handholding was preferred over stroking (χ2(1) = 54.921, p < .001), whereas the difference between stroking and handholding in non-intense situations was not significant (χ2(1) = 3.236, p = .072).*

**Discussion
- Although well-written, the first part of the discussion goes too far. All the studies were online and featured videos and hypothetical situations. Therefore, the authors need to be clear that these were not actual, real, in-person situations, where the results could be different.**

As suggested, we now state explicitly in the first paragraph that the situations referred to were hypothetical/recalled.

*The overall pattern of results confirmed our hypotheses. Handholding was preferred over stroking, and in almost all studies that preference was stronger in hypothetical and recalled situations judged to be intense. Handholding was stronger in emotional than in physical situations, though to a lesser extent. It was preferred by both recipients and providers and was strongly preferred behaviorally by partners of women giving birth. These results suggest that, at least in hypothetical and recalled situations, participants subjectively preferred handholding over stroking as a type of touch suitable for regulation of acute emotions and pain.*

**- Near the bottom of p. 24, the authors say, ‘…as opposed to stroking, which relies mainly on bottom-up processes’. I do not fully agree that stroking specifically relies mainly on bottom-up processes. Although receiving stroking is passive touch, the situation will determine the perception, including various top-down effects. Such top-down effects may be more relevant for active touch in hand-holding, but they are present in both situations.**

We agree that the previous wording was imprecise. The sentence now reads: “The emotional regulation effects of handholding may be the result of top-down processes, as opposed to stroking, in which bottom-up processes may play a larger part”.

**- In the middle of p. 25, the authors say, ‘even if handholding is found in the future to be less
effective than stroking’, but what does this mean? Less effective in what way? I am not sure if I follow all the section on ‘effectiveness’, as this concept has not been well-defined throughout. Do you mean how effective touch is to console/comfort someone?**

We appreciate this comment and have rephrased the paragraphs using the term “effectiveness” to clarify that refer to effectiveness in regulating emotions (i.e., comforting):

*Finally, subjective preference might be influenced by actual differences in their emotion regulation effects. As we detailed above, it could be the case that by allowing for two-way communication [4], handholding is a better method of homeostatic emotional regulation [3]. However, this possible mechanism must be corroborated with future research on emotion regulation effectiveness.*

## *Limitations and implications for future research*

*First, the current study focused on subjective preference for types of touch. As we detailed in the introduction, research on the actual effectiveness of touch of both types of touch is inconclusive, and more research on such effectiveness is still needed. However, our findings do provide strong evidence that people prefer handholding as a form of emotion regulation, which is important regardless of its actual emotion regulation effects: even if handholding is found in the future to be a less effective method of emotion regulation than stroking, its effects should still be examined as our findings show that it is much more likely to be performed in real life situations involving intense emotions.*

**Minor comments
- p. 14, ‘hypothesize themselves in different hypothetical situations’ could be revised for clarity. Imagine themselves?**

We appreciate this correction and have made the appropriate correction in manuscript.

1. *As we experimentally assigned each participant to see the exact same number of intense and non-intense situations, we did not test for an intensity main effect. The slight difference in the number of situations (389 vs 395) is due to us switching the classification of two of the situations post randomization, as detailed above. The significance of none of the results in this section changed due to the switch.* [This footnote appears in the manuscript text as well]
