# Supplementary material for: I wanna hold your hand: Handholding is preferred over gentle stroking for emotion regulation
Source: PLoS One. 2023 Apr 6;18(4):e0284161. doi: 10.1371/journal.pone.0284161 (PMC10079127; doi:10.1371/journal.pone.0284161)
Supplement: Attachment — Submitted filename: Reviewer response final.docx [file pone.0284161.s004.docx]

We have addressed all of the reviewers’ comments, and our response to each specific point is included below.

While working on the revised version, we found a small coding error in the second part of Study 2 (response provision) – for some of the participants, touch ratings of 5 were instead encoded as 6. We updated all of the analyses with the updated data; none of the main results, regarding preference for handholding over stroking or its interaction with intensity has changed (i.e., significant results becoming non-significant or vice versa). The results that did change are:

- Previously, all touch types were rated significantly higher in negative physical situations. Now, this effect is not significant in the second part of Study 2 when using dichotomous measures. This effect does not involve specific touch types and as such is tangential to the paper.
- Previously, handholding was preferred more in negative situations in the second part of Study 2 when using dichotomous measures; now, this effect is not significant. This effect was already ruled as unstable, as it was not found using continuous measures, was not found at all in the first part of Study 2 and a reversed effect was found in the second part of Study 1. Thus, it was not and is still not discussed.
- The simple slope for stroking in intense situations is now significantly positive in the second part of Study 2 when using continuous measures – stroking was preferred more in intense situations than in non-intense situations. The simple slope for handholding is still larger and the interaction is still significant and in the same direction, and as such this does not impact our main findings.

Finally, after the correction, the ordinal analyses did not converge with a fully saturated model on the second part of Study 2 when using continuous measures. They did converge when removing the random slopes for touch types (but keeping random slopes for intensity and for the interaction of intensity and touch type), with the same effects being significant – this is the model that we report in the paper. This is detailed in a footnote:

*The fully saturated cumulative link model did not converge for the second part of Study 2 (touch provision) when using participant intensity ratings (the model using dichotomous situation classifications converged successfully). Thus we ran that model without a random slope for touch type (but with random slopes for intensity and the interaction between intensity and touch type); this was the only way to have a converging model while removing only one random slope. We performed additional robustness tests on this specific study which are detailed in the S1 appendix.*

The additional checks listed in the appendix are:

*As the fully saturated cumulative link model for the second part of Study 2 did not converge when using participant-rated intensity, we performed additional robustness checks. First, we ran an intercept-only ordinal model which converged and demonstrated identical significant effects to all other analyses. We also determined that the convergence issues were due to data from a single participant (participant 952408). Running the fully saturated model without this participant converged with identical significant effects to all other analyses. To ensure that the effects were not artifacts caused by removing this participant’s data, we also ran a fully saturated model with this participant while artificially increasing one of this participant’s ratings for stroking in one of the intense situations from 1 to 3 (i.e. weakening both of the significant effects, which state that ratings for stroking are lower than for handholding, especially in intense situations); This analysis also converged with identical significant effects to all other analyses.*

**Reviewer #2: Although the authors have made a significant effort to revise their manuscript, important issues have not been resolved. Moreover, I am concerned that their statements are too broad and unjustified by the data. They send the wrong message and may do more harm than good for the field. Below I explain my main theoretical/conceptual issues and then elaborate on some of the outstanding methodological/analytical problems.

I previously asked that the authors introduce the distinction between reciprocal and non-reciprocal touch in their introduction when discussing differences between handholding and stroking and the bidirectionality of the former, which is lacking for the latter. They wrote in their response that there is no research about this. However, this distinction has been around for decades. I suggest looking at the book by Hall and Knapp (**[**https://www.amazon.com/Nonverbal-Communication-Human-Interaction-Knapp/dp/1133311598**](https://www.amazon.com/Nonverbal-Communication-Human-Interaction-Knapp/dp/1133311598)**). Again, I consider this distinction central to the comparison they make and something that should be raised when introducing their research question. Ultimately, their handholding condition is active, whereas their stroking condition is passive and it is more than simply tactile processes that differ here. There are additional motor and proprioceptive processes that contribute to hand-holding. There is also the issue that simply holding onto something (be it another person or another object) could help regulate discomfort or pain.**

As suggested, we have added a discussion of reciprocity to the introduction:

*The importance of handholding as a bidirectional or reciprocal form of touch can also be viewed in the broader context of reciprocity in nonverbal interaction[25]. In general, people are motivated to reciprocate others’ actions if those actions create pleasant arousal [26]. For example, one study found that couples are more likely to reciprocate each others’ touch the more established their relationship is [27]. The use of handholding in emotion regulation is somewhat distinct from classic reciprocity as defined in the literature as the touching person is not reciprocating the touched person’s action (i.e., they are not initiating handholding in response to touch by the other person); instead, they are responding to the other person’s emotion in a manner which, in the case of handholding, has a reciprocal nature. To the best of our knowledge there is no study comparing reciprocal and non-reciprocal types of touch in the context of emotion regulation. Still, there could be a general positive reaction to reciprocity; for example, in one study [28] people who performed reciprocal forms of touch are more highly regarded than people who perform non-reciprocal forms of touch.*

We also address active/passive touch and holding onto an object as possible mechanisms in the discussion:

*Finally, other possible mechanisms include differences between active and passive touch and the regulation effects of holding an object (not necessarily a hand). Regarding active and passive touch, to the best of our knowledge no existing study examined differences in their emotion regulation capacities. However a review of differences in their sensory capabilities found studies pointing to either type of touch as better performing [59]. The authors suggest that the differing factor may be task complexity, with passive touch performing better in sensing simple (as opposed to complex) stimuli. It could be the case that another person’s skin is not an overly complex stimulus, and as such the passive touch involved in handholding might perform better. Additionally, in both handholding and stroking the touch recipient is engaged in passive touch, limiting differences in this regard to the touch provider.*

*Regarding the effects of holding an object, it could theoretically be the case that the effects of handholding are merely the effects of holding an object. However, while holding an object (e.g., a squeeze ball) is a recognized form of distraction in the medical literature, it is not significantly different than other forms of distraction such as watching cartoons or hearing music [60,61]. Thus, there is no specific reason to believe that stroking would not generate similar distraction. Indeed, emotion regulation studies directly comparing holding an object to handholding find larger effects for handholding, suggesting that additional mechanisms are at play [9,62].*

**I was now able to view the gif files. One thing I noted is that stroking seemed significantly faster than 3 cm/s. Perhaps time changed in the process of creating the gifs? Can the authors measure the speed in the images and document that?**

We now report that:

*The touch literature specifies exact parameters for optimal stroking – stroking of the arm using the index and middle fingers, at speeds of 1-10 cm/s [11,38,39]. As describing this optimal procedure could confuse participants, videos were used instead of verbal labels to depict the different types of touch (Fig 1). The videos showed only the actors’ hands against a flat white background. The videos depicted presence (i.e., two hands not touching each other), stroking, and handholding. In the video depicting handholding the actors were instructed to hold their hands statically. In the stroking videos, the actors were asked to stroke their partner’s arm covering an area of nine cm over a period of three seconds. The videos were then evaluated by judges to confirm that they look natural. Follow-up inspection of videos revealed a range of speed between 4-12 cm/s.*

**In general, the stroking looks a bit mechanical rather than affectionate. Moreover, I don’t think the position of the people stroking is typical for the manner in which people stroke each other. When would you stroke someone’s forearm standing side by side? It is uncomfortable. This position is much more natural for handholding. For several of the clips the action is not in center and moves out of the frame. There are also significant shadows of the body parts on the walls that could affect perception.**

This was also noted by reviewer 3 after the previous version. We agree that the fact that we had a limited number of videos is a limitation of the study and could have introduced additional biases. It has been addressed in the limitation section, and we now explicitly mention shadows:

*Third, as detailed above, the study used videos to present optimal stroking without biasing participants with an overly detailed description of stroking compared to handholding. Of course, while the videos were carefully designed to reduce possible biases – by using neutral backgrounds, not showing faces, etc. - some characteristic of the videos which was not controlled for might still have affected results. Some possible factors include video quality (e.g., non-steady camera, shadows), the fact that the actors were all in opposite-sex relationships while participants could have been of any sexual orientation (and in any type of relationship), or the fact that both types of touch were depicted in the air (i.e., not sitting down, or close to the body). This is somewhat mitigated by the first part of Study 4, in which types of touch were presented verbally, without videos, and still “stroking” was overwhelmingly less common as a form of support for women giving birth than “handholding”.*

**I wonder whether body part matters. Could a rub on the back be more comfortable/preferred than rubbing of the forearm? In any case, I think that a generalization to all kinds of stroking is not possible here. The authors don’t compare hand-holding with stroking, they compare hand-holding with forearm stroking (from an unnatural position with a certain speed).**

This is also a point raised by reviewer 3 after the previous version. We specifically mention that by “stroking” we refer to forearm stroking. Once in the abstract:

*Stroking (specifically of skin with C-tactile afferents on the forearm, i.e. C-touch)*

And in a footnote:

*“Stroking” can mean many things – stroking can be performed in many different parts of the body at various speeds and forms. As the current study aims to match effectiveness studies, unless explicitly stated otherwise, we use “stroking” to mean using one’s hand to stroke another person’s forearm at a speed of 1-10 cm/s, the optimal speed found in most studies.*

**I had asked previously that more information about the situations in Study 1 and 2 be provided. I have not found this in the revision. I’m wondering what kind of emotions were examined (e.g., sadness, anger, fear)? Could stroking be more effective for low-arousal states such as dejection and handholding for high-arousal states such as fear? Indeed, I’m skeptical that the data allow the authors to conclude that emotion regulation in general benefits more from handholding than stroking. Emotion regulation is a very broad class of processes that includes the enhancement, maintenance and reduction of any kind of emotion.**

Regarding information about the situations, we are not sure what information is missing as the full text of all situations, as well as intensity and valence statistics for all of them, was already provided in the appendix. To avoid further confusion, the information is reproduced here below this response.

Regarding generalization, while our study was not equipped to test every emotion by itself (e.g., to separate anger, sadness and fear), it did test a variety of emotional situations, including high and low arousal situations, and we could find no consistent condition in which stroking was preferred over handholding. Thus, we believe that the generalization is merited. While we have already called for additional research to find specific situations in which stroking is preferred, we have expanded on that call to note the limits of the correct study:

*On the other hand, while the current study examined a variety of situations, it could not explore every possible one. More subjective preference research should be conducted to investigate whether there are situations – involving emotion regulation or otherwise – in which people prefer stroking over handholding.*

Information about situations from the appendix:

Situations

Below are listed the situations provided in the study (Table S5), as well as their mean intensity and valence ratings (Table S6). Spearman correlations between mean ratings of situations in each study showed that the situations were ranked extremely consistently between the studies (.985 for intensity and .994 for valence).

Table S5

|  | *Physical Negative* | *Emotional Negative* | *Physical Positive* | *Emotional Positive* |
| --- | --- | --- | --- | --- |
| *Intense* | *A: You are at the dentist's clinic. Imagine that you have just undergone a painful dental procedure. (1)*  *B: You are at home. Imagine that you have just fell down some stairs and landed on your knees. (2)* | *A: You are at home. Imagine that you are about to give an online presentation to a large audience. You are not sure you have prepared enough and are very anxious. (5)*  *B: You are at home. Imagine that you have just received news that you have failed an important test. (6)* | *A: You are at a dance party. Imagine that one of your favorite songs has come up. It's a fast and exciting song and you are dancing intensely. (9)*  *B: You are at home. Imagine that you're eating food you brought from a great restaurant and it tastes amazing.* (10)* | *A: You are at home. Imagine that you were just notified that you've won 300$ on a bet. (13)*  *B: You are climbing a hill on a short trip. Imagine that you are seeing a beautiful landscape on the top of the hill.* (14)* |
| *Non-Intense* | *A: You are at home. Imagine that you have caught a cold and are not feeling well. (3)*  *B: You've eaten something bad and are resting at home. There are no severe symptoms, but you generally feel a bit nauseous and tired. (4)* | *A: You are at home. Imagine that you have just been informed that you have lost 20$ on a bet. (7)*  *B: You are at home. Imagine that a person close to you is about to move to a faraway country for a long time. (8)* | *A: You are at home. Imagine that you've just finished getting dressed after taking a nice shower and you're feeling refreshed. (11)*  *B: Imagine that you've just woken up on a weekend morning after having a great night's sleep.* (12)* | *A: You are at home. Imagine that you have just gotten home after a great day at work / school. (15)*  *B: You are at home. Imagine that you have just read a heartwarming article about someone who overcame a harsh disease and went on to do amazing things.* (16)* |

** These situations’ intensity was originally coded differently for Study 1 (intense situations were originally coded as non-intense and vice versa).*

*Table S6*

| *Situation #* | *Classification* | *Intensity* | | *Valence* | |
| --- | --- | --- | --- | --- | --- |
|  |  | *Study 1 mean(SD)* | *Study 2 mean(SD)* | *Study 1 mean(SD)* | *Study 2 mean(SD)* |
| *1* | *Physical, negative, intense* | *3.23(.67)* | *3.22(.81)* | *1.89(1.08)* | *2.07(1.4)* |
| *2* | *Physical, negative, intense* | *3.09(.73)* | *3.15(.75)* | *1.71(.87)* | *1.83(1.07)* |
| *3* | *Physical, negative, non-intense* | *2.11(.68)* | *2.11(.72)* | *2.3(.67)* | *2.72(1.23)* |
| *4* | *Physical, negative, non-intense* | *2.21(.56)* | *2.2(.75)* | *2.23(.71)* | *2.6(1.05)* |
| *5* | *Emotional, negative, intense* | *3.06(.7)* | *3.1(.76)* | *2.53(1.12)* | *2.74(1.35)* |
| *6* | *Emotional, negative, intense* | *3(.67)* | *3.07(.73)* | *1.64(.65)* | *1.91(1.25)* |
| *7* | *Emotional, negative, non-intense* | *1.61(.64)* | *1.66(.68)* | *2.78(.68)* | *3.09(.91)* |
| *8* | *Emotional, negative, non-intense* | *2.94(.83)* | *2.99(.71)* | *2.12(.92)* | *2.36(1.37)* |
| *9* | *Physical, positive, intense* | *2.72(.73)* | *2.64(.83)* | *6.05(1.02)* | *5.94(.84)* |
| *10* | *Physical, positive, intense* | *2.22(.91)* | *2.23(.87)* | *6.15(.8)* | *6.09(.86)* |
| *11* | *Physical, positive, non-intense* | *1.52(.76)* | *1.68(.88)* | *5.81(.81)* | *5.57(.85)* |
| *12* | *Physical, positive, non-intense* | *1.48(.85)* | *1.71(.92)* | *6.05(.91)* | *5.88(.99)* |
| *13* | *Emotional, positive, intense* | *2.81(.73)* | *2.86(.74)* | *6.5(.76)* | *6.21(1.18)* |
| *14* | *Emotional, positive, intense* | *2.54(.79)* | *2.51(.83)* | *6.31(.77)* | *6.12(.94)* |
| *15* | *Emotional, positive, non-intense* | *1.84(.76)* | *2(.869)* | *5.97(.8)* | *5.89(.84)* |
| *16* | *Emotional, positive, non-intense* | *2.07(.84)* | *2.02(.821)* | *5.85(1.03)* | *5.79(.95)* |

*Figure S1*

**Relatedly, it seems from the discussion that some of the situations were positive? Did participants opt for handholding to increase/maintain positive and/or reduce negative emotions? Much of the paper is written in a way that suggests touch is used to down-regulate negative feelings but it seems the data may not clearly speak to that and may be more nuanced.**

As detailed in the manuscript, interaction analyses found no consistent touch type*valence interactions, even though our valence measures show that we have covered a wide range of valence. Thus, our findings hold equally well for both increasing/maintaining positive and reducing negative emotions. The discussion is somewhat weighted toward regulating negative emotions, as the emotion regulation literature shows that in everyday life the vast majority of emotion regulation instances involve negative emotions (Gross et al., 2006). We now note this in the discussion:

*Exploratory findings for valence and physicality are summarized in Table S7 in the S1 appendix. Focusing on moderation of touch type preference by valence and physicality, most effects were only significant in a single part of a single study, including three-way interactions and moderation of handholding preference by valence. Thus, the results support the notion that the effects are not limited to positive or negative-valenced situations. While our discussion is somewhat focused on regulation of negative emotions, which are the vast majority of emotion regulation instances reported in everyday life [59], our findings demonstrate that handholding is equally preferred to regulate either type of emotion.*

Gross, J. J., Richards, J. M., & John, O. P. (2006). Emotion Regulation in Everyday Life. In D. K. Snyder, J. Simpson, & J. N. Hughes (Eds.), Emotion regulation in couples and families: Pathways to dysfunction and health (pp. 13–35). American Psychological Association. [https://doi.org/10.1037/11468-001](https://psycnet.apa.org/doi/10.1037/11468-001)

**So with all that’s said above, it is not appropriate to say that handholding is more effective than stroking for emotion regulation. Instead, the authors should carefully reflect on their methods/results and offer a more nuanced discussion and summary of findings in their title, abstract and main body of the paper.**

As suggested, we now tone down our conclusions and do not claim that handholding is more effective than stroking for emotion regulation:

*In conclusion, the results of four studies demonstrate a consistent subjective preference for handholding over stroking as a form of emotional regulation, especially in intense situations. We suggest several possible explanations for this phenomenon, including the cultural familiarity of handholding, top-down processes having stronger effects than bottom-up processes on subjective preference, handholding being a more passive form of touch, and handholding’s unique capability to establish two-way sensory communication, creating an optimal feedback loop.*

We hope that this change, alongside our response to the specific points raised by the reviewer addresses this general concern.

**Other concerns:
Page 14: „Continuous Situation Intensity and Valence“ I had mentioned in my previous review that these are not continuous measures.**

We have completely removed the mention of “continuous” variables from the paper and from the appendix. **I’m unclear about what the authors mean by intensity? How is it different from being very positive or negative? How were participants instructed to do the rating?**

The Miriam-Webster dictionary defines intensity as “the quality or state of being intense; especially **:** extreme degree of strength, force, energy, or feeling”. This is what we think of when we refer to intensity – a situation which elicits an extreme degree of feeling. This is similar to being “very positive” or “very negative”, as an extreme degree of positive emotion is “very positive” and an extreme degree of negative emotion is “very negative”. Still, there are some additional distinctions. For example, a situation in which a strong negative outcome is diffused over time may be less intense than one in which a less negative outcome is experienced in a very specific moment (see for example that situation 3 – catching a cold – was rated as or more negative than situation 5 – being anxious before giving a presentation – but was rated much less intense).

This relation – being similar but not identical to “very positive” or “very negative” – is also borne out in the data. The zero-order correlations between intensity and valence in both parts of studies 1 and 2 ranged from 0.29 to 0.42 in positive situations and from -0.6 to -0.38 in negative situations, suggesting a strong link but not identical constructs.

Still, the current paper does not aim to be a psychometric paper on the nature of intense emotions; as such, we do not aim to demonstrate correlations between the concept of “intensity” and other constructs, and instead stick to the word “intensity”.

This matches instructions given to participants, which were simply asked how intense did each situation feel, as we now explicitly note:

*Each participant rated the intensity of each situation by responding to the prompt “How intense does this situation feel?” on a 4-point Likert scale ranging from “not intense at all” to “very intense”. They also rated the valence of each situation by responding to the prompt “How positive or negative does this situation feel?” on a 7-point Likert scale ranging from “very negative” to “very positive”.*

As we noted above, participants’ intensity ratings of situations were extremely consistent between studies (spearman correlations > .96 between mean intensities of situations in each study), suggesting that participants understood this concept similarly to one another.

**The logistic regression should include random intercepts and slopes. I’m also confused about the choice of test here. It would seem a chi-square test would be more appropriate. This is indeed what is used in Study 4.**

The logistic regression was already redone with random intercepts and slopes for the previous revision. As with all proposed statistical analyses results were identical. This was previously in the appendix but has now been moved to the main text. Regarding chi-square tests, assuming the reviewer is referring to the first part of Study 1, we have also already analyzed these data in the previous version using chi-square tests, and that analysis has also now been moved to the main text.

**I had asked previously that the rating analyses be revised in line with the statistical properties of the data. I don’t understand why the revised analyses are in the supplementary materials rather than the main body of the manuscript? Also, the analyses there are inappropriate. To analyze the ratings, the authors should run an ordinal model with all random slopes and intercepts, rather than an ordinal model with only random intercepts and a linear model with random intercepts and slopes, which is what they have now placed in the supplementary materials. Tables S10 and 11 show no degrees of freedom. They are also missing in the main text.**

The ordinal models were indeed run with random intercepts and slopes, this is now stated explicitly (with the caveat noted above in the opening of the response letter, regarding the second part of Study 2):

*These include modeling random slopes for all variables, using cumulative link models[37] implemented in the R package ordinal [38] (also with random slopes for all variables), and using simple repeated ANOVA tests to test effects with dichotomous intensity ratings.*

The cumulative link models provide z values and not t values as was erroneously stated – we now note that they are z values, which do not involve specific values of degrees of freedom. We have also moved the tables to the main text.

**Follow-up tests of the interaction between intensity and touch were not reported. The authors say what package they used, but do not explain what they actually did. Did they use the simple_contrast function on their LME model? For the results, the reader is sent to the Appendix.**

We now explicitly note that the function used was simple_slopes. We have also moved the table to the main text (Table 3). **The figures have not improved. The authors count data points and participants without discriminating between the two. So they don’t present subject means and do not allow readers to get a sense of the variance in the data. There are a couple of rain cloud plots, but these are not fully presented. It seems the distributions were very broad and are cut off at the margin of the figure.**

All figures in the main text are now violin plots of subject means. The fact that the distributions are cut off is due to the fact that the scales used in the study are bounded and that participants use extreme points of scales (even when using means, subject means are means of at most 4 ratings, and some subjects use the extreme point of the scale for all 4 ratings in a given cell).

To allow interpretation of within-participant variance, we have also added figures denoting each data point to the appendix; in these figures the y axis denotes specific participants such that a single participants’ data can be viewed by looking along a horizontal line:

***Full Data Plots***

***Figure S2.*** *Study 1 touch ratings per participant by touch type and intensity*

*Participants are arranged along the y axis such that specific heights correspond to specific participants. Participants are sorted that participants with high mean ratings for handholding are at the top. Touch ratings indicate the way participants thought their feelings would change if they received that type of touch.*

***Figure S3.*** *Study 2 touch reception ratings per participant by touch type and intensity*

*Participants are arranged along the y axis such that specific heights correspond to specific participants. Participants are sorted that participants with high mean ratings for handholding are at the top. Touch ratings indicate the way participants thought their feelings would change if they received that type of touch.*

***Figure S4.*** *Study 2 touch provision ratings per participant by touch type and intensity*

*Participants are arranged along the y axis such that specific heights correspond to specific participants. Participants are sorted that participants with high mean ratings for handholding are at the top. Touch ratings indicate the way participants thought their partners’ feelings would change if they received that type of touch.*

***Figure S5.*** *Study 3 touch ratings per participant by touch type and intensity*

*Participants are arranged along the y axis such that specific heights correspond to specific participants. Participants are sorted that participants with high mean ratings for handholding are at the top. Touch ratings indicate the way participants thought their feelings would change if they received that type of touch.*

***Figure S6.*** *Study 4 touch ratings per participant by touch type and intensity*

*Participants are arranged along the y axis such that specific heights correspond to specific participants. Participants are sorted that participants with high mean ratings for handholding are at the top. Touch ratings indicate the way participants thought their feelings would change if they received that type of touch.*

**Page 30: „physical or emotional – on a 1-100 scale, with no verbal indicators for
endpoint“ How can the authors be certain that all participants used this scale as intended by them? I could consider ratings as reflecting the amount of pain that is being reduced or the amount that pain is being reduced to.**

We now cite the prompt word by word:

*For example, for physical pain the prompt was “For each of the following types of touch, please rate on a scale of 1 to 100 how much did it help reduce your physical pain during the contractions”.*

The prompt states that the number refers to the amount of “help” (i.e., high numbers mean that touch is useful). While we agree with the reviewer that the question might have been phrased better, the clear main effect which matches the findings of Studies 1-3 as well as the other part of this study suggests that the question was adequate.

**Page 32: „was strongly preferred behaviorally by people assisting women giving birth“ This was not examined. You simply examined the kind of touch women giving birth recall having received from their partner.**

We have now added to the sentence “(as recalled by the women)”.

**I looked at the OSF archives. I could only find some MTURK pilot data that was the same for Study 1 and 2. Otherwise I found no data.**

It seems that the reviewer was looking at files attached to the preregistration which obviously do not include the final data, which did not exist at the time. The link already provided in the paper to the full data is:

<https://osf.io/nju48/?view_only=81dadc972f6545f19d107c83d8dba368>

where all of the data was already available inside Open Analysis.zip which also includes an R project which runs all of the analyses. To avoid further confusion, we have now added to the same project a folder labeled “data” which includes all of the data from all of the studies with no need to extract a zip file.

**Reviewer #3: Authors have addressed all my comments.

However, I have some comments on the revised version:
- Please make sure you proofread the paper as some grammatical errors are present (e.g. "was significantly differed")**

We have performed an additional proofreading pass.

**- Some comments seem to not be included in the main text; while mentioned in the response to reviewers, please justify / change it. For example for experiment 1, authors states that they elaborated the chi-square results "beyond asking participants to rate ..." but I cannot find this paragraph in the revised version of the manuscript.**

The specific paragraph noted by the reviewer was in the appendix; it has now been moved to the main text. We have examined all of the comments mentioned in the previous and current responses to reviewers and all now appear in the main text, except when it was explicitly noted that they appear in the appendix.

**- On that note, the second part of this added paragraph is not totally clear to me. "Touch type chosen and intensity were not statistically independent (χ2(2)= 43.317, p < .001), meaning that handholding was preferred in intense situations." Could you please reformulate/correct ?**

We have rephrased the sentence, which now reads:

*Touch type chosen and intensity were significantly associated with one another beyond chance (i.e., not statistically independent; χ2(2) = 43.317, p < .001), meaning that handholding was preferred in intense situations.*

**- Figure legends: could you expand the legends, especially for Fig1 and 2, explaining what are the images picturing? For fig 2: are these the 4 types of touch participants rate in all studies ? Add this info in the figure legend. For fig 1, is the first image picturing stroking or no touch ?
For figure 6 to 9 what does the legend "touch rating" represent ? please explain in the legend and on the figure as "touch rating" is not specific enough. Please be clearer on what is presented, as for now figures are difficult to understand.**

We have added captions for all figures and meaningful labels for specific touch rating levels and intensity labels, including listing the specific types of touch depicted in Figures 1 and 2.

**- Study 2: why not include the chi-square analyses results in the descriptive part ?**

We assume the reviewer is referring to Study 1 which was the only one in which we conducted Chi-Square analyses. These analyses were moved to the main manuscript; as the descriptives are shared for the first and second parts of the study, we included the Chi-square analyses when discussing the first part of the study, where they are relevant.

**- Page 31: touch reception ratings in study 4: which figure are you referring to? Probably figure 9. On that note, why representing the results differently here from the other studies ?**

Thanks for spotting this – we have now inserted the correct figure number. The results of this study are represented differently as the touch ratings in this study have 100 levels instead of 5, such that a full representation of each level as in the other studies is unfeasible.

**- Overall, I would advise to go again over the results section of each study to improve clarity.**

We have performed another clarity pass on the results.

**Reviewer #4: The Authors have addressed my concerns to a satisfactory extent, and I believe that the manuscript is now suitable for publication.**
