# Supplementary material for: I wanna hold your hand: Handholding is preferred over gentle stroking for emotion regulation
Source: PLoS One. 2023 Apr 6;18(4):e0284161. doi: 10.1371/journal.pone.0284161 (PMC10079127; doi:10.1371/journal.pone.0284161)
Supplement: Attachment — Submitted filename: Reviewer Comment Revision 2 Final.docx [file pone.0284161.s003.docx]

We wish to thank the reviewers for their comments, and detail our responses below.

**Reviewer #2: This is a very interesting and potentially important study. However, there are too many open issues concerning both the study methods and analyses that make it impossible to clearly evaluate the actual value of the data. I list below the things that require further explanation and re-analysis.

Page 3: The idea of allostasis was already implemented in early models of homeostasis. Now, the term allostasis is typically used to refer to deviations that have some sort of clinical implication or are disease relevant. Apart from this issue, I don’t quite see the usefulness of introducing the concept of allostasis as it is not needed for the paper. Indeed all that’s needed is the idea of a feedback loop.**

We thank the reviewer for this comment and have now removed the term in the introduction; we still briefly note it in future directions for readers who are familiar with its use in Fotopoulou and colleagues (2021) which is one of the main theoretical bases of the current paper.

**Page 4: In the last paragraph, the critical distinction is that handholding enables reciprocal touch whereas stroking does not. So the classical distinction between reciprocal and non-reciprocal touch really is central here and should be introduced accordingly.**

We wish to thank the reviewer for this comment. Indeed, the main issue introduced here is the distinction between reciprocal and non-reciprocal touch. We have examined the literature on reciprocal and non-reciprocal touch, but unfortunately most of the research we could find that involves this distinction does not compare reciprocal to non-reciprocal touch. We now include one study that made a somewhat relevant direct comparison in the discussion:

*While we could find no direct comparison of top-down effects, one study [52] explored difference in the ways people perceive reciprocal types of touch, which include handholding, as opposed to non-reciprocal types of touch, which include stroking (although these two specific types of touch were not directly examined in the study). The study showed that people regarded both participants in reciprocal touch slightly higher (on dimensions of valence and likability) than providers of non-reciprocal touch, and significantly higher than recipients of non-reciprocal touch. Thus, people thinking about providing or receiving handholding might be thinking about themselves as more likeable and with a more positive valence, which might lead them to choose this type of touch over others regardless of its actual bottom-up effects.*

**Page 8: There is no value in doing post-hoc power analyses. This should be deleted.**

We have deleted these analyses.

**Page 9: I found no touch videos following the link. I only found a zip archive with photos and it wasn’t clear to me how they were being presented. Their names contained numbers and letters that were little intuitive. As this is the key manipulation, what exactly was shown needs to be better explained and illustrated in the main manuscript. Just from looking at Figure 1 it would seem that there was a lot more skin contact for the hand holding condition than for the stroking condition and that would constitute a confound. I believe this was addressed in Study 2 but this isn’t explained or visually supported in any way.**

Concerning the visualization in the document: Figure 2 now shows all four types of touch, and the section on “touch label videos” now explains that the full stroking video was added in Study 2.

Concerning the files on OSF: the videos were provided in GIF format (which is the format through which users saw it in their web browsers). While the study was presented through the web, ensuring that the files would be displayed as videos, some operating systems default to opening GIF files with picture editing programs which show each frame as a separate photo. We added a file which describes the contents of the OSF archive, which reads:

*Description of project files:*

*OpenAnalysis.zip contains an R project which runs all of the analyses used in the paper.*

*HandGifs.zip contains the videos used in studies 1-3 in GIF format. The “Full Stroking” video, showing stroking done with the whole hand instead of only two fingers, was only used in studies 2 and 3. Two versions of each video were created, marked “Set A” and “Set B”. Each user saw a randomly chosen set of videos in each part of the experiment.*

*Note that some operating systems open GIF files as a series of photos (one photo for each frame of the video), opening the GIF with a web browser should show the video.*

**More information about the situations is needed. Please plot means and CIs for ratings for each of the selected situations. Report the rating scale endpoints. As suggested below, it would be useful to do this for each study separately using the study participant data rather than the data obtained in the pre-experimental rating.**

As suggested, we now include a mean and CI plot for situations (Figure S1 in the S1 appendix), as well as numerical values (Table S6, there), separated by study. Endpoints for all scales are reported in the methods section:

*Each participant rated the intensity of each situation on a 4-point Likert scale ranging from “not intense at all” to “very intense”, and the valence of each situation on a 7-point Likert scale ranging from “very negative” to “very positive”.*

(…)

*In the second part of the study, touch preference was measured more granularly by comparing participants’ ratings of each type of touch. Participants read the following instructions: “Please rate how would that kind of touch affect your feeling?” and responded on a 5-point Likert scale ranging from “would make me feel much worse” to “would make me feel much better”. Types of touch were presented using looping videos (Fig. 2– note that this figure includes an additional type of touch which was only added in Study 2).*

**Page 11: Please report the software/tool you used to do your power analysis. This is not trivial in the context of mixed modeling.**

We now report that we used the R package paramtest to perform simulations.

**Page 13: Its a bit disconcerting that intensity flip flops between different samples. This suggests that it is not a stable measure and likely not useful to test your hypotheses. In your results you report analyses using the participant’s own ratings as a variable. Given these issues that would be the more reasonable way to go. So I would do away with the pre-experimental ratings and trying to categorize situations into intense and not intense. Your stats is a bit dense as it is. No need to add to this by including analyses that aren’t informative… Note that this also raises concerns about how reliable/consistent your valence and physicality ratings were. Why would we trust those? Again, I would suggest dropping those. As a reader I am not convinced…**

The changes in intensity were between a small pilot sample which rated a longer list of situations and the actual study samples which are much larger. Following the reviewer’s previous suggestions, we now include full data on mean valence and intensity per study for the various situations. To assess the difference between the samples, we performed a spearman correlation analysis between the mean rating of each situation in each sample. As reported in the S1 appendix:

*Spearman correlations between mean ratings of situations in each study showed that the situations were ranked extremely consistently between the studies (.985 for intensity and .994 for valence).*

As the pre-experimental ratings were preregistered, we feel that removing them completely from the paper might be confusing. However, following the reviewer’s suggestion, we have attempted to reduce discussion of them even further and relegate more of it to the appendix.

Regarding valence, as described above the ratings were highly consistent. Regarding physicality, physicality analyses are only included in the manuscript as they were mentioned as exploratory analyses in the preregistration. However, we now discuss the issue of physicality in the limitation section:

*Second, in Studies 1 and 2 situations were classified as physical or emotional (non-physical). We did not ask each participant to code situations on this axis, and as such cannot verify the validity of this classification. However, the physicality classification has no bearing on our main hypotheses concerning touch preference and intensity*

**The reader is variously referred to S1 appendix and supplementary materials. Are they the same? I could only find S1 appendix with this submission. But some of the information (e.g., situation intensity ratings for the different studies) was not provided.**

All of the mentions of supplemental materials have been removed, we now refer only to the S1 appendix. We have also added situation intensity ratings for each study.

**Page 15: Here we learn for the first time that there are two different stroking conditions. This is a bit confusing. I also don’t understand how the videos were assigned to situations. What exactly were the “video sets”? Again this is the key manipulation and needs to be fully explained.**

**The description of the task is a bit misleading. What you say in the text and what is shown in Figure 1 are at odds. If I understand Figure 1 correctly, then participants did not select what type of touch condition they preferred. Instead, they rated for each touch condition, how it would make them feel in a given situation.**

We wish to thank the reviewer for pointing out that our description of the task was not clear enough. As part of our restructuring of the whole methods section, we now clearly explain that the videos were simply used instead of labels for the ratings questionnaires. We refrain from using the term video sets which we agree is confusing, and now simply detail that videos were recorded by two couples, as well as the way we used them in the study:

*In the first part of the study, touch preference was measured using a direct choice. Participants were asked “Which kind of touch would you prefer to receive from your partner in that situation?” and selected one of the types of touch on the screen. Types of touch were presented using looping videos (Fig. 1).*

*In the second part of the study, touch preference was measured more granularly by comparing participants’ ratings of each type of touch. Participants read the following instructions: “Please rate how would that kind of touch affect your feeling?” and responded on a 5-point Likert scale ranging from “would make me feel much worse” to “would make me feel much better”. Types of touch were presented using looping videos (Fig. 2– note that this figure includes an additional type of touch which was only added in Study 2).*

***Figure 2. The touch rating screen shown to participants.***

***Touch Label Videos***

*The touch literature specifies exact parameters for optimal stroking – stroking of the arm using the index and middle fingers, covering an area of nine cm over a period of three seconds [11]. As describing this optimal procedure could confuse participants, videos were used instead of verbal labels to depict the different types of touch (Fig 1). The videos showed only the actors’ hands against a flat white background. The videos depicted presence (i.e., two hands not touching each other), stroking, and handholding. In the video depicting stroking, the actors were asked to stroke their partner’s arm using the optimal procedure described above.*

*Videos were recorded by two heterosexual couples who had been in their romantic relationship for over a year. Each couple recorded one video for each type of touch. Videos by one couple were used in the first part of the study, and videos by the other couple were used in the second part, chosen randomly for each participant.*

We have also depicted these two types of questions separately in new Figures 1 and 2, which also show how participants selected a type of touch in the first part of Study 1.

**Page 16: A 5-point Likert scale is not a continuous measure. It is an ordinal measure and should be treated as such. I recommend the ordinal package in R.**

We thank the reviewer for introducing us to the R ordinal package. Following this remark, we have used the ordinal package to re-run all of the main analyses – touch type main effect and touch x intensity interactions - in Studies 1-3 (Study 4 uses a 1-100 slider). All results remained the same. We report this analysis in the manuscript:

*To ensure that the effects reported here are not due to specific statistical choices, we have re-run analyses for the main hypotheses using different statistical methods. These include modeling random slopes for all variables, using cumulative link models[37] implemented in the R package ordinal [38], and using simple repeated ANOVA tests to test effects with dichotomous intensity ratings. All of the results stayed the same – no significant effect became non-significant and vice versa.* *Results for these analyses are provided in the Robustness Tests section in the S1 appendix.*

Still, we thought that fully reporting on multiple ways of performing each analysis in the main manuscript would be cumbersome. The manuscript still reports specific statistics from the original analyses for three reasons. First, these analyses were preregistered. Second, the debate whether ordinal measures are necessary is still ongoing (e.g., Wu & Leung [2017] show that ordinal measures are highly [ > .9] correlated with continuous ones). Finally, Likert scales are overwhelmingly analyzed using continuous measures (At least until 2017, 100% of the papers in three leading journals exclusively used continuous measures [Liddell & Kruschke, 2018]). It was reasoned that using a new analysis in a paper that is not methodologically focused might confuse readers.

Liddell, T. M., & Kruschke, J. K. (2018). Analyzing ordinal data with metric models: What could possibly go wrong?. *Journal of Experimental Social Psychology*, *79*, 328-348.

Wu, H., & Leung, S. O. (2017). Can Likert scales be treated as interval scales?—A Simulation study. *Journal of Social Service Research*, *43*(4), 527-532.

**Please report the software and packages you used to conduct your statistical analysis.**

We now cite using R and the various software packages we used.

**How did you convert what you call “preference rating” (which is a misnomer) into numbers? This is necessary to understand the data in Table 1. Later sections of that table are non-legible due to line breaks. There are variables in there that haven’t been introduced (e.g., UCLA). The table really isn’t helpful. I suggest presenting key information in figures, and place a cleaned up version of the table in your supplementary materials. Current figure 2 just shows the models not the data. Please find a proper way to plot your data. I suggest rain-cloud plots for their transparency (**[**https://www.r-bloggers.com/2021/07/ggdist-make-a-raincloud-plot-to-visualize-distribution-in-ggplot2/**](https://www.r-bloggers.com/2021/07/ggdist-make-a-raincloud-plot-to-visualize-distribution-in-ggplot2/)**).**

We thank the reviewer for this suggestion, and now include plots of intensity and touch type preference by situation intensity for each study. We used raincloud plots for the z-score and 1-100 values in Study 4 (Figure 9); for Studies 1-3 the values were discrete so we plotted them using simple histograms (Figures 4,6-8). As the following reviewer asked for more information in tables, we opted to keep the tables in the manuscript.

**So I assume you had 8 values for each cell per participant on average? If you don’t model random slopes, you are increasing your type 1 error. If you enter more than one value per cell per participant you should add random slopes or include situation as a factor. Alternatively, if these models don’t converge, as you are not interested in situation you should use a mean across situations. Also please report missing cells as a function of subjects. I would assume there are some. One would hope there are not too many including cases that rate each situation equally intense…**

As suggested, we re-ran the analyses with random slopes for all variables and none of the results changed (see tables S8 and S9).

Regarding missing data, we appreciate the reviewer pointing us to be more explicit on this issue. In Studies 1-3 only participants with full data were included in the analyses. For Study 1, after re-examining our data following the reviewers’ comment, we found that two participants excluded for missing data actually did provide all of the data needed for the current study (they were excluded as they did not click a “submit” button at the end of the study). The main text still reports the same dataset including 99 participants which was preregistered, but we have run all of the main analyses including the two additional participants with no meaningful changes to the results. We note this in a footnote:

*Two additional participants were erroneously removed from the dataset before pre-registration as they made a technical error while submitting their questionnaires. We re-ran all analyses including these participants and all results remain the same. Results for these analyses are provided in the Robustness Tests section in the S1 appendix.*

For Study 2, all participants provided full data. For Study 3, we have added details on missing data to the existing footnote:

*Two participants who completed very few items were erroneously not mentioned in the pre-registration document. All analyses used only the 51 participants for whom full data is available. Three of the remaining participants coded one out of three situations and one coded two out of three situations.*

The small number of participants with missing data in this study does not allow for any meaningful analysis of their characteristics, especially as all of them except one rated only one situation such that we could not compare their intensity ratings between situations.

In Study 4 we have added more detail regarding missing data:

*All participants answered questions concerning recalled touch and concerning intensity and the effects of touch on physical pain during contractions, and concerning the intensity of physical pain between contractions. 7 participants quitted the survey at that point, leaving 29 participants who answered questions about touch effects on physical pain between contractions. One participant quitted the study at that point, leaving 28 participants who answered questions on intensity and touch effects on emotional pain during contractions. 9 participants quitted the survey at that point, leaving 19 participants who completed the final questions on emotional pain between contractions.*

And in a footnote:

*We could not find any meaningful differences between participants who quit early and participants who did not. The main effect found for touch (higher ratings for handholding over stroking) was found even when looking only on touch effects on physical pain during contractions, where data was available for all participants.*

Regarding the reviewer’s specific concern about intensity ratings being equal in missing data, here are the intensity z-score means for intense situations (in this study, during contractions) and non-intense situations (in this study, not during contractions). As can be seen, for all subgroups intensity in intense situations is higher than intensity in non-intense situations. For subgroups 1,2,and 4 the means are significantly different; subgroup 3 included only one participant so a t-test could not be performed but that participant’s intensity ratings are more different than the mean differences of all other groups:

|  | N | Intense situation mean intensity z-score (SD) | Non-intense situation mean intensity z-score (SD) | Difference t-test |
| --- | --- | --- | --- | --- |
| 1. Full data | 19 | .292(.97) | -.328(.932) | t(73.9) = 2.82, p = .006 |
| 2. Quit after rating intensity of physical pain between contractions | 7 | .482(.492) | -.591(.841) | t(9.7) = 2.8, p = .019 |
| 3. Quit after rating the effect of touch on physical pain between contractions | 1 | .6(N/A) | -1.25(N/A) | N/A |
| 4. Quit after rating the effect of touch on emotional pain during contractions | 9 | .524(.762) | -.542(.921) | t(13.5) = 2.92, p = .011 |

**Earlier in the manuscript you state that you tested both positive and negative situations and different types of what you call physicality. What happened with these manipulations in your analyses? This goes back to the issue that its not clear what exactly was presented in terms of combinations of the situations and touch types.**

All of these analyses were present in the appendix. Following this comment, we now summarize all of these analyses in the result section of each study and discuss key findings in the discussion section. Full tables are still in the appendix.

Concerning the procedural aspects of these variables, we have completely revised the methods section, and we hope that the new explanation makes clear which situations were presented:

***Procedure***

*All studies were conducted online via the Qualtrics survey platform. Participants began by providing informed consent and indicating the length of their romantic relationship. In the first part of the study, participants were asked to imagine themselves in 8 hypothetical situations in which their romantic partner touched them in order to make them feel better. For each situation, participants rated its intensity and positivity/negativity. They were then asked to pick one type of touch. The available types of touch were presence (i.e., no touch), handholding, and optimal stroking. To ensure that the participants understood which type of touch is discussed, the types of touch were not labelled with words (e.g., “handholding”), but instead with looping 5-second videos showing the type of touch (see details below).*

*In the second part of the study, participants saw 8 different situations, and similarly rated intensity and positivity/negativity. However, this time they were asked to provide a rating for each type of touch. Again, the types of touch were labelled with looping videos. As detailed below, each participant saw the same 16 situations which were assigned randomly to one of the two parts of the study. The touch labels were presented in random order.*

*After these two parts, they completed a loneliness questionnaire for a separate project.*

***Measures***

***Situations***

*A list of hypothetical situations was generated using a pilot study. We showed 21 participants a list of 33 emotional or physical situations generated by the researchers and asked them to rate the valence and intensity of each situation. For each context (emotional or physical), we selected the two situations rated the most positive and intense as the positive intense situations for that context. We similarly selected three additional pairs of situations (negative intense, positive not intense, negative not intense). This process yielded 16 situations classified as intense/not intense (intensity), positive/negative (valence), and physical/emotional (context). Two situations were available for each of the eight possible configurations of these variables. In the actual study, two positive situations originally designated as intense (one physical and one emotional) were each rated less intense than one of the corresponding non-intense positive situations. Thus, the designations of these two situations were switched.*

*In each part of the study, participants saw 8 situations. Situation order was randomized such that in each part of the study participants saw one situation of each designation (e.g., one physical, non-intense, negative situation; one physical, non-intense, positive situation, etc.).*

**Please report how you determined effect sizes from your mixed models. This is a bit tricky for LMEs.**

We now detail this in the manuscript:

*All analyses are accompanied by partial f^2^ effect sizes, calculated using the procedures outlined by Selya and colleagues[36]. To calculate f^2^ for a specific predictor, R^2^ was calculated for the complete model (R^2^_Total_), and­ for the model without the predictor (R^2^_Omitted_); f^2^ was calculated as (R^2^_Total_ - R^2^_Omitted_) / (1 - R^2^_Total_).*

**How did you follow-up interactions? Did you do this within the full model or did you run simpler separate models? Did you look at the intensity effect for each level of touch? Please spell things out for your reader.**

We now state in the manuscript that:

*We performed simple slope analyses via contrasts using the R package reghelper[39].*

We have looked at intensity effects for each level of touch as detailed in the manuscript, for example for Study 1:

*Ratings for handholding significantly increased with situation intensity. Ratings for stroking did not significantly change with intensity (when using dichotomous intensity classification, ratings for stroking significantly decreased with intensity).*

**Page 25: Now here you really do look at preference. Please be clear throughout your manuscript what your items actually measure.**

We thank the reviewer for pointing this out. We have reviewed all of the appearances of the word “preference” in the manuscript, to clarify that preference is a construct which we measure in two ways – by asking participants to perform a direct choice (in the first parts of Study 1 and Study 4) and by comparing participants’ ratings of the types of touch (in all other studies). This is discussed explicitly in the methods section for Study 1:

*Touch Preference*

*In the first part of the study, touch preference was measured using a direct choice. Participants were asked “Which kind of touch would you prefer to receive from your partner in that situation?” and selected one of the types of touch on the screen. Types of touch were presented using looping videos (Fig. 1).*

*In the second part of the study, touch preference was measured more granularly by comparing participants’ ratings of each type of touch. Participants saw the prompt “Please rate how would that kind of touch affect your feeling?” and responding on a 5-point Likert scale ranging from “would make me feel much worse” to “would make me feel much better”. Types of touch were presented using looping videos (Fig. 2– note that this figure includes an additional type of touch which was only added in Study 2).*

**I’m unclear what you did with the logistic regression. It would seem that you treated data points within participants as independent. That would be inappropriate. How can you have a significant touch effect when touch preference (handholding vs stroking) was your DV? Or what was your DV? More information is needed.**

The logistic regression was a mixed model to account for within-participant dependencies. We now provide the explicit equation for the logistic regression:

*The direct touch type choices performed in the first part of the study were analyzed in a mixed logistical regression using the R package lme4 [27], using the following equation:*

$${Choice}_{ij} \sim b_{0}+b_{1i}+b_{2}* {Intensity}_{ij}$$

*The choice of participant i in situation j – stroking or handholding was estimated using a fixed intercept (b_0_), a random intercept for each participant (b_1i_), and an intensity effect (b_2_). Occasions on which participants chose presence over the other types of touch were not analyzed.*

As with all of the mixed regression models, we repeated the mixed logistic regression with random slopes for all variables (i.e., intensity) and the effects did not change.

As for the effects, the DV was indeed the type of touch. This means that the intercept indicates which type of touch was chosen more, and the intensity slope indicates how this effect changed with intensity. This is now spelled out in the results section:

*The results confirmed our hypothesis that handholding would be chosen significantly more often stroking (i.e., the intercept was positive and significant; b = .329(SE= .096), z = 3.427, p < .001) and that this effect would be stronger in more intense situations (i.e., an intensity effect, b = .593(SE = .101), z = 5.847, p < .001).*

**For study 4, did you specifically ask about hand holding and hand stroking? What if there was other stroking touch that didn’t target the hand? That would seem to be a problem with interpreting the frequency of handholding vs stroking.**

We appreciate this remark. If the frequency of stroking was higher or equal to that of handholding, this could have presented a problem with interpreting the results. As the frequency of stroking was significantly lower, this only means that the effect might be even stronger. We now state this in the manuscript:

*Out of 36 participants, 7 (19.4%) reported that the helping person stroked them, while 25 (69.4%) reported that the person held their hand. To confirm our hypothesis that handholding would be more prevalent than stroking, we performed a chi-square for goodness of fit test comparing the actual distribution between handholding and stroking (19.4% and 69.4%) with a chance distribution (50% for each type of touch). The test revealed that the distribution was significantly differed from chance (χ^2^(1) = 10.125, p = .001). Importantly, “stroking” was not explicitly specified to be on the hand or forearm. Thus, out of the 7 participants who reported receiving stroking, some may have received stroking in other parts of the body, making the difference in prevalence between handholding and stroking of the hand or forearm even larger.*

**The results section ends with reports on valence and physicality effects. As mentioned earlier I’m unclear how these variables intersect with what else was presented in each study. No information is provided about how they were statistically analyzed. Hence, results cannot be evaluated.**

Tables with full results of all of the analyses concerning these variables are provided in the appendix. We currently briefly state in the main manuscript that:

*In exploratory analyses with additional independent variables, fixed slopes were included for each main effect and for every possible interaction.*

The equation for the valence and physicality analysis, which currently does not appear in the manuscript, is:

$${{Rating}_{ij} \sim b_{0}+b_{1i}+b_{2}* {Intensity}_{ij}+b_{3}* {Touchtype}_{ij}+b_{4}* {Valence}_{ij}+ b_{5}*{Physicality}_{ij}+ b_{6}* {Intensity}_{ij}* {Touchtype}_{ij}+ b}_{7}* {Intensity}_{ij}* {Valence}_{ij}+ b_{8}* {Intensity}_{ij}* {Physicality}_{ij}+ b_{9}* {Touchtype}_{ij}* {Valence}_{ij}+ b_{10}* {Touchtype}_{ij}* {Physicality}_{ij} + b_{11}* {Valence}_{ij}* {Physicality}_{ij}+ b_{12}* {Intensity}_{ij}* {Touchtype}_{ij}* {Valence}_{ij}+ b_{13}* {Intensity}_{ij}* {Touchtype}_{ij}* {Physicality}_{ij}+ b_{14}* {Intensity}_{ij}* {Valence}_{ij}* {Physicality}_{ij}+b_{15}* {Touchtype}_{ij}* {Valence}_{ij}* {Physicality}_{ij}+ b_{16}*{Intensity}_{ij}* {Touchtype}_{ij}* {Valence}_{ij}* {Physicality}_{ij}$$

We believe that the brief explanation above is sufficient for exploratory analyses that did not yield important findings, but are happy to add the full equation (as well as parallel equations for the other exploratory analyses) to the manuscript at the editor’s discretion.

**I am not reading the discussion as I don’t see much point. There simply are too many question marks concerning the paradigm and analyses. In my opinion this needs another major revision in which the methods and result sections are improved. The results should include only appropriate analyses that are methodologically justified and properly explained.**

We hope that the complete restructuring of the methods and results section, as well as our changes in response to the reviewer’s previous comments, are enough to address this concern.

**Reviewer #3: In this four-experiments study, authors aimed to show that handholding was a stronger social touch than stroking to regulate intense situations. This is an interesting and very relevant study – however I have several comments that could be taken into account in order to improve the manuscript.

Overall, it is not clear why the authors chose handholding and stroking over other types of social touch. Especially in the abstract stating that “social touch highlights two forms of touch: handholding and stroking”, is quite limiting (and not true – see for ex Hertenstein et al. 2009; McIntyre et al. 2022). Authors should rephrase and discuss limitations accordingly. And in the introduction, page 4 please back up your discourse by references when stating that there are two main forms of consoling touch.**

We appreciate this comment, and have made some changes to clarify in what sense these types of touch are central – namely, that they were previously studied in the context of the effectiveness of touch as a form of emotion regulation. The abstract line quoted by the reviewer was replaced with:

*In recent years, the emotion regulation effects of two types of touch have been studied extensively: handholding and gentle stroking.*

At the very beginning of the paper, we now explicitly mention other functions of touch (such as the work on emotion communication detailed by the reviewer) and explain that the current manuscript is focused on touch as a form of emotion regulation.

*Touch is an important form of interaction in humans as well as in other species [1]. Interpersonal touch serves a variety of functions, including emotion communication[2] and modulating interpersonal bonds[3]. The current study focuses on touch as a form of emotion regulation: one major reason humans touch one another is to help regulate subjective experiences such as negative emotions and pain, sometimes referred to as consoling or comforting touch.*

Finally, we have removed the sentence noted by the reviewer, and replaced it by specifically mentioning that these types of touch have been chosen due to their prominence in effectiveness research.

*Two types of touch have been thoroughly examined in recent years using traditional paradigms testing the effectiveness of touch in distress regulation: handholding and gentle stroking.*

**Moreover, there seems to be a general bias towards the authors hypothesis of preference for handholding in general: indeed, by looking at the gif presented to the participants, the naturalness of the handholding is much stronger than for the stroking. Authors could try to control for this (asking new participants to rate naturalness) and/or add it as a strong limitation. On the same line, the stimuli picturing the stroking conditions are not very well filmed (camera moving) and it is rare to perform this type of touch on the move / in the air. Moreover, there are quite a lot of different types of stroking so authors should acknowledge this.**

We thank the reviewer for this important comment. We have now added a limitation discussing the fact that we had a small number of video sets which could have introduced additional biases:

*Third, as detailed above, the study used videos to present optimal stroking without biasing participants with an overly detailed description of stroking compared to handholding. Of course, while the videos were carefully designed to reduce possible biases – by using neutral backgrounds, not showing faces, etc. - some characteristic of the videos which was not controlled for might still have affected results. Some possible factors include video quality, the fact that the actors were all in opposite-sex relationships while participants could have been of any sexual orientation (and in any type of relationship), or the fact that both types of touch were depicted in the air (i.e., not sitting down, or close to the body). This is somewhat mitigated by the first part of Study 4, in which types of touch were presented verbally, without videos, and still “stroking” was overwhelmingly less common as a form of support for women giving birth than “handholding”.*

**The study by Burgoon 1991 is interesting however indeed they did not test arm stroking, so authors should rephrase their conclusion/reformulate as arm touching is quite different to arm stroking (stroking hasn’t been shown to communicate dominance but more comfort). Also, slow vs fast stroking does not communicate the same emotions (see for ex Kirsch et al. 2018**).

We appreciate the reviewer’s comment. Following previous notes by the reviewer, we tried to make sure that the paper focuses on touch as a form of emotion regulation, leaving touch as emotion communication out of the scope. As such, we have removed the discussion of the Burgoon paper from the manuscript. **I would advise to refrain from talking about C-touch for slow caress like stroking, just use the slow gentle stroking or caress like stroking terminology.**

We generally agree with this comment. However, as many existing studies use the term C-touch, we felt that avoiding it completely could confuse readers. In the only two places we use the term, we explicitly say that this is a term others use to refer to this type of touch. We also explain why we avoid using it in a footnote:

*Recent studies have found some C-tactile afferents in the palm skin as well, albeit at a much lower density [12]; other studies show that these afferent can be activated by deep pressure and not only be stroking, which might mean that they are activated by handholding as well [13]. As such, we avoid using the term C-touch for stroking in the rest of the manuscript as both types of touch might be activating C-tactile afferents.*

**In the introduction – before the overview – it would be nice to justify the reason why you are investigating “touch provision vs reception, situation valence, emotional vs physical and cultural differences”. Moreover, actually you are not testing cultural differences (or results missing).**

We have added more details on our choice of moderators:

*Beyond exploring our main hypothesis regarding preference, we examined several possible moderators. First, we examined whether the hypothesized preference for handholding would be stronger in more intense situations. The homeostatic regulation model led us to believe that if two-way sensory communication is indeed crucial for regulating acute disturbances in homeostasis, handholding may be more effective, and therefore preferred, as a way to regulate emotions in intense situations. Thus, we hypothesized that the preference for handholding would be stronger in intense situations.*

*Second, we examined in a more exploratory manner whether situation valence (i.e., whether the experience is positive or negative) and physicality (i.e., whether the experiences involved physical as opposed to purely emotional pain or pleasure) would moderate the effect. Effectiveness studies have examined the effects of touch in both positive and negative contexts (e.g., pleasantness [15] versus pain [19]), and when regulating both emotional [9] and physical [28] pain, but to the best of our knowledge have not compared these contexts to one another. As such, we did not have a specific directional hypothesis concerning valence and physicality.*

*Third, Study 3 aimed to examine whether including a situation that was specifically relevant to participants – namely, an injection for participants with blood/injection phobia – would induce a different pattern of results. We examined whether the level of phobia would moderate results.*

*Finally, as we could not find effectiveness studies which performed cultural comparisons, we sought to examine whether touch preference would be different in different cultures.* *To do so, in Study 4 we compared the touch preferences of Arab and Jewish women. Importantly, these last two moderators – blood/injection phobia and culture – build on less established literature. As such, they were only included in one study each (Studies 3 and 4, respectively), and our investigations of them are intended to inform future research, rather than to reach solid final conclusions.*

As for the (null) results concerning cultural differences, they were previously reported in the appendix, but have now been moved to the main text (full tables are still in the appendix).

**Authors should justify from the introduction the reason why they are conducting the studies only in people being in a heterosexual romantic relationship. And then discuss the implied limitations.**

The study was not limited to heterosexual couples, as participants could have been of any sexual orientation and surveys were explicitly worded not to assume partners of specific genders. It is true that the three couples who recorded videos were heterosexual. We believe this is highly unlikely to have affected results as the videos did not include gender signifiers, but we now mention this as a limitation as part of our discussion of the limited number of videos.

*Third, as detailed above, the study used videos to present optimal stroking without biasing participants with an overly detailed description of stroking compared to handholding. Of course, while the videos were carefully designed to reduce possible biases – by using neutral backgrounds, not showing faces, etc. - some characteristic of the videos which was not controlled for might still have affected results. Some possible factors include video quality, the fact that the actors were all in opposite-sex relationships while participants could have been of any sexual orientation (and in any type of relationship), or the fact that both types of touch were depicted in the air (i.e., not sitting down, or close to the body). This is somewhat mitigated by the first part of Study 4, in which types of touch were presented verbally, without videos, and still “stroking” was overwhelmingly less common as a form of support for women giving birth than “handholding”.*

**In study 4 – authors state that ‘in cases where they were not provided a particular type of touch..’: how often does this happened? As it is very different to rate received vs hypothetical touch.**

We appreciate this comment and have added it as a limitation. As we detail in the limitation, the low number of women who received stroking and\or did not receive handholding does not allow us to make empirical conclusions regarding this issue based on the current sample:

*Relatedly, in the second part of Study 4 some of the participants received the types of touch they were rating during their actual childbirth experience, while some did not, and were rating them hypothetically, a difference which may have affected the results. As only 7 women (under 20%) received stroking and only 11 women (less than 35%) did not receive handholding, analyzing these subgroups would be underpowered. That said, Studies 1-3 conclusively show that even when all situations are hypothetical, handholding is preferred over stroking.*

**For the touch preference rating: this section is very unclear. Please clarify what the participants had to do: rate each video or choose the one they preferred and rate only this one? Probably the terminology used is confusing: as it is not a preference rating but how it would make people feel. Please reformulate.**

We have completely reformulated our discussion of these ratings and reviewed every use of the word “prefer” in the manuscript to clarify that preference is a construct measured either by direct choice or by comparing ratings:

*In the first part of the study, touch preference was measured using a direct choice. Participants were asked “Which kind of touch would you prefer to receive from your partner in that situation?” and selected one of the types of touch on the screen. Types of touch were presented using looping videos (Fig. 1).*

***Figure 1. The touch preference screen shown to participants****.*

*In the second part of the study, touch preference was measured more granularly by comparing participants’ ratings of each type of touch. Participants saw the prompt “Please rate how would that kind of touch affect your feeling?” and responding on a 5-point Likert scale ranging from “would make me feel much worse” to “would make me feel much better”. Types of touch were presented using looping videos (Fig. 2– note that this figure includes an additional type of touch which was only added in Study 2).*

***Figure 2. The touch rating screen shown to participants.***

**For data analyses: why coding 0,5 vs -0,5 the type of touch, and not use a categorical variable? Moreover, could you have run simple ANOVA on your data? Please better justify you analyses.**

Using a categorical variable essentially dummy codes binary variables as 0 and 1. In that case, other effects in the equation that do not include the variable in question reflect the effect when the categorical value is a specific value (e.g., the main intensity effect would reflect the intensity effect for stroking). By using a balanced dummy coding main effects of one variable are interpretable as effects across categories of other variables (e.g., the main intensity effect reflects the average intensity effect across touch types). This is now stated in the manuscript:

*Touch type was coded 0.5 for handholding and -0.5 for stroking, so that coefficients for other effects would reflect average effects across the two touch types.*

As for ANOVA, the analyses reported in the main text had continuous independent variables (intensity) and as such we obviously could not use ANOVA. The analyses using dichotomous codings of intensity, which are included in the appendix, use linear mixed models to be comparable to the analyses using continuous variables. Still, we conducted ANOVA analyses wherever we could, with no meaningful change in results. This is detailed in the appendix:

*Other Analyses. In all other cases, when preforming analyses using dichotomous situation classifications instead of continuous ratings, dichotomous classifications were dummy coded 0.5 and -0.5 (to allow for the interpretation of effects as effects that average over categories of other values). Even though the use of repeated measures ANOVA was theoretically possible, we used linear mixed models, to preserve equivalence with analyses using the continuous ratings. Still, to test robustness, we performed ANOVA analyses wherever possible; no meaningful difference in results was found.*

**It is unclear what is the difference between table p18 and table p20? Why including UCLA ratings there is you are not analyzing it? Moreover, it is unclear in these tables how you take into account valence (positive vs. negative scenario).**

The table on p18 was a descriptive table, while the table on p20 was analysis results. The confusion might have been due to the fact that the coefficients were labeled “mean” instead of “b” – this has now been fixed. We have removed descriptives for UCLA. As for valence, we feel that providing eight versions of each descriptive – one for each level of the three (2 valence levels x 2 intensity levels x 2 physicality levels) would be excessive. Of course, analyses of the effects of valence are provided in the appendix.

**Table 2 is unclear: if it represents the results of the mixed linear model could you please add the b, CI and R2? Also as in the text you are mentioning slopes p24 but they are not reported.**

All tables now include 95% CIs. b values were included but were labelled as “Mean”, they are now labelled as “b”. As the previous reviewer has noted, there is no single standard way to calculate R^2^ values from mixed models. The method we use to calculate effect sizes outputs f^2^ values which are included in the tables:

*All analyses are accompanied by partial f^2^ effect sizes, calculated using the procedures outlined by Selya and colleagues[29]. To calculate f^2^ for a specific predictor, R^2^ was calculated for the complete model (R^2^_Total_), and­ for the model without the predictor (R^2^_Omitted_); f^2^ was calculated as (R^2^_Total_ - R^2^_Omitted_) / (1 - R^2^_Total_).*

We intentionally do not include whole model R^2^ values – these values are inflated as they include the variance explained by random intercepts and by control variables.

Simple slopes analyses are now fully reported in the S1 appendix, Tables S12 and S13. **Please add in the Statistical Analysis section all the other analyses you have run: how you analyzed the subjective preference, recalled perception etc.. As for example the chi-square analyses are unclear.**

The statistical analyses section has been expanded to separately detail the use of logistic regression for analyzing direct choice and mixed linear regression for analyzing ratings. We wish to thank the reviewer for pointing us to the chi-square analyses for recalled touch in Study 4, as the way we were using chi square for independence there was incorrect. We now use chi square for goodness of fit, as detailed below (results did not meaningfully change):

*Out of 36 participants, 7 (19.4%) reported that the helping person stroked them, while 25 (69.4%) reported that the person held their hand. To confirm our hypothesis that handholding would be more prevalent than stroking, we performed a chi-square for goodness of fit test comparing the actual distribution between handholding and stroking (19.4% and 69.4%) with a chance distribution (50% for each type of touch). The test revealed that the distribution was significantly differed from chance (χ^2^(1) = 10.125, p = .001).*

As for testing the direct choice task in Study 1 using dichotomous measures which employed chi-square tests (as all variables are categorical), we have elaborated on the description of statistical methods:

*Results for analyses on Study 1. Beyond asking participants to rate each touch type, we also asked them to choose a single type of touch they would want to receive the most. First, we performed a Chi-square test for goodness of fit to compare the overall number of times each type of touch was chosen (349 times handholding, 251 times stroking) to a chance (equal) distribution. The test confirmed that the distribution was significantly different from chance, meaning that handholding was significantly preferred over stroking overall (χ2(1) = 16.007, p < .001). We then performed a chi-square test for independence between the type of touch chosen and situation intensity. Touch type chosen and intensity were not statistically independent (χ2(2) = 43.317, p < .001), meaning that handholding was preferred in intense situations. We tested simple effects by comparing the distribution of touch type choices to chance, separately for intense and non-intense situations. Tests for simple effects found that in intense situations handholding was chosen more than stroking beyond chance (χ2(1) = 54.921, p < .001), whereas the difference between stroking and handholding in non-intense situations was not significantly different from chance (χ2(1) = 3.236, p = .072).*

**Figures in general should be improved (titles, axes, legends). For example, in figure 2 what is ‘improvement’? The same scales should be used in all the graphs to be comparable; and in the legend please indicate what are the scales - for example why is intensity represented from -2 to 2, when the participants answer on a 4-point Likert scale?**

We have improved labelling on all figures. We now use identical scales except for Study 4, where the study itself used different scales (and there we note that intensity is a z-score, as original ratings were 0-100).

**Discussion:
It is unclear how results support this statement “It is much more likely to be performed in real life situations involving intense emotions”. Please justify and clarify.
Please add all limitations mentioned in points above.**

The whole discussion has been reformatted and the sentence mentioned has been removed. As detailed in the response to each point, all limitations have been added.

**Reviewer #4: It was a pleasure to able to review this manuscript titled: I wanna hold your hand: handholding is preferred over gentle stroking for acute emotion regulation. I have read the manuscript with great interest as it (supposedly) addresses an open question in the field of social touch: what types of touch are more suited to provide emotional support. I found the study interesting in principle and well powered. I really appreciate the large sample size and the fact that all the studies were preregistered. However, I found the process of reading and understanding the paper very challenging. I provide more specific comments below, but generally my main concerns are regarding the introduction and the discussion that should be completely revised, in my opinion. Too many generalizations are made, working hypotheses are cited as evidence, and scientific rigor is lacking. Furthermore, the present studies are not grounded in the literature, the results are very difficult to follow, the discussion does not do what a discussion is meant to do, which is to discuss the results in the context of what was already known in the field. Overall, I have noticed a lack of clarity and precision throughout the manuscript, and the conclusions are not fully supported by the results. Therefore, I am not in the position to recommend acceptance of the manuscript in the present form. I am providing some suggestions that could potentially help to improve the manuscript in turn below:

• Abstract: It is not clear from the abstract that the studies were conducted online. This should absolutely be included in the abstract, otherwise it can be misleading.**

The abstract now states that the studies were conducted online.
 **• Page 5: On the second page of the introduction, it remains unclear whether “consoling touch” should be classified as a touch that targets homeostatic or allostatic regulation. I think it is important to clarify this, or to say that consoling touch can address both needs…?**

Following the suggestion of a different reviewer, we have mostly removed mentions of allostatic regulation from the manuscript. Consoling touch is also a term that we find too non-specific for our purposes, and as such we only note it briefly as it is sometimes used in other papers. **• Page 5: When talking about the positive effects of handholding and touch more generally, I think it is important to stress the fact that such effects are modulated by individual differences in patterns of relating (e.g., attachment). Furthermore, there is a large body of studies that are very relevant and have just been neglected here. Please see below:

Krahé, C., Paloyelis, Y., Sambo, C. F., & Fotopoulou, A. (2014). I like it when my partner holds my hand: Development of the responses and attitudes to support during pain questionnaire (RASP). Frontiers in Psychology, 5, 1027.
von Mohr, M., Kirsch, L. P., & Fotopoulou, A. (2017). The soothing function of touch: affective touch reduces feelings of social exclusion. Scientific reports, 7(1), 1-9.
von Mohr, M., Krahé, C., Beck, B., & Fotopoulou, A. (2018). The social buffering of pain by affective touch: a laser-evoked potential study in romantic couples. Social cognitive and affective neuroscience, 13(11), 1121-1130.
Krahé, C., von Mohr, M., Gentsch, A., Guy, L., Vari, C., Nolte, T., & Fotopoulou, A. (2018). Sensitivity to CT-optimal, affective touch depends on adult attachment style. Scientific reports, 8(1), 1-10.
Krahé, C., Paloyelis, Y., Condon, H., Jenkinson, P. M., Williams, S. C., & Fotopoulou, A. (2015). Attachment style moderates partner presence effects on pain: a laser-evoked potentials study. Social cognitive and affective neuroscience, 10(8), 1030-1037.
Krahé, C., & Fotopoulou, A. (2018). Psychological and neurobiological processes in coping with pain: The role of social interactions. In The Routledge International Handbook of Psychobiology (pp. 73-92). Routledge.
Burleson, M. H., & Quigley, K. S. (2021). Social interoception and social allostasis through touch: legacy of the somatovisceral afference model of emotion. Social neuroscience, 16(1), 92-102.**

We appreciate the reviewer pointing us to these interesting papers, and now cite them in the manuscript.

**• Page 6: “ …stroking is uniquely associated with elevated oxytocin levels (13)”. I invite the Authors to be more cautious about this. I would advise to edit this sentence as to clarify that this is a working hypothesis. (e.g., ….it has been suggested that …).**

We appreciate this correction. We have revised our citation of this paper, which now reads:

*It has also been suggested that stroking may be associated with elevated oxytocin levels [21].*

**• Page 6: When saying that handolding and C-touch types of touch are effective forms of emotion regulation, I would like to think this is true and it is something that many labs have been trying to prove, but the authors must be more precise and should avoid mentioning this as a fact. Also, which types of emotions are they referring to? Citing some papers to support this statement would be helpful.**

As suggested we now cite some relevant papers to the statements regarding the effectiveness of handholding and C-touch in emotion regulation.

**Updated after continuing reading: But then, when I continue reading, I find the paragraph saying that “existing studies in the field have focused on testing the effectiveness of touch as a method of emotion regulation, with most studies …”. So, the Authors now question something that was given as certain a few lines above? Perhaps this section should come first, or the Authors might want to be a bit more clear here.**

We have amended the first statement to clarify that we consider the aforementioned studies as evidence for the claim:

*The studies cited above have demonstrated many cases in which both handholding and stroking manage to regulate emotions (creating a sense of pleasantness, and reducing pain, anxiety and feelings of social inclusion)*

And have clarified the second statement:

*The existing studies in the field detailed above have demonstrated the effects of handholding and stroking separately. However, studies comparing the effectiveness of one of these types of touch with the other are scarce, with studies comparing two types of touch often comparing stroking of the forearm to stroking or touching of the hand, but not to handholding.*

**• Page 6: “…as opposed to stroking, handholding allows both participants to sense each other equally and simultaneously using their hands, which are more effective as tactile sensory organs than the forearms”. I find sentences like this one quite problematic for the following reasons:
1. The sensory organ in touch is always the skin. The hands and the forearms are not sensory organs, they are body sites.
2. Stroking can also refer to palm against palms, and as such, the proposed difference between handling and stroking would not apply anymore.
3. Stroking can also be delivered from forearm to forearm, and as such both individuals would be equally and simultaneously touched
4. Touch is always a two-way sensory communication, so I find this statement very weak as a basis for the hypothesis of this study.
5. To me, one of the main differences between stroking and handholding (that is not mentioned at all) has more to do with the fact that handholding is static, while stroking is a dynamic type of touch.**

1. We have removed the word “organ”, which is indeed technically incorrect in this context.
2. We have added a footnote clarifying that as we are trying to match effectiveness studies which focus on the forearm, when we discuss stroking we refer to stroking as tested in those studies, which is always on the forearm:

*“Stroking” can mean many things – stroking can be performed in many different parts of the body at various speeds and forms. As the current study aims to match effectiveness studies, unless explicitly stated otherwise, we use “stroking” to mean using one’s hand to stroke another person’s forearm at a speed of 3 cm/s, the optimal speed found in most studies.*

3. Again, while forearm on forearm touch is theoretically possible, to the best of our knowledge it is not studied in effectiveness studies and as such was not examined here.
4. We thank the reviewer for this note, which encouraged us to clarify our argument: two-way communication is always possible, but in handholding (compared to hand-on-forearm or forearm-on-forearm stroking) both participants engage their fingers and palms which have much more tactile sensitivity than the forearm, potentially allowing for much better communication:

*While any form of touch allows for two-way sensing, the tactile sensitivity of the palms and fingers is larger by orders of magnitude than that of the forearms [22,23].*

5. This idea did not occur to us before running the studies and as such did not influence our pre-registered hypotheses. Given the results showing a preference for handholding, this does not seem to explain the results, as we would assume that if anything people would prefer a dynamic type of touch.

**• Page 6: “….studies compared general pleasantness of touch between touching arm and hand skin found little difference (18).” The Authors are citing the meta-analysis of Cruciani et al in an incorrect way, for what I understood. Cruciani and colleagues discussed differences between hairy and non-hairy skin. Here the Authors discuss differences between arm and hand. The hand has both hairy (dorsal part of the hand) and non-hairy skin (palms), so I think the Authors should double check and be consistent with the use of terms throughout the manuscript, otgherwise it is all very confusing.**

We have reexamined the meta-analysis by Cruciani and colleagues, which clearly state (p. 91, second paragraph):

*As displayed in Fig. 2, the meta-analysis did not show a significant difference between pleasantness ratings in response to affective stimulations delivered on the forearm with respect to the palm (18 studies; 464 subjects; Hedges’ g = 0.010, 95 % CI [-0.16, 0.18], p = 0.875).*

And in the discussion (p. 91, first paragraph of discussion):

*No systematic preference towards affective stimulations on the forearm (i.e., hairy skin) or on the palm (i.e., glabrous skin) was observed across the studies*

To be as clear as possible, we have switched the word, “arm” to “forearm” and “hand” to “palm”, and the sentence now reads:

*Studies that compared general pleasantness of touch between touching forearm and palm skin found little difference [25].*

**• Generally, in the introduction, the Authors do not mention various, very important pieces of knowledge in the field of social touch that can be very relevant to build the framework for this paper:
1. CTs can be found also on the non-hairy skin of the body (e.g., palms); this body part is involved in handholding: Watkins, R. H., Dione, M., Ackerley, R., Backlund Wasling, H., Wessberg, J., & Löken, L. S. (2021). Evidence for sparse C-tactile afferent innervation of glabrous human hand skin. Journal of Neurophysiology, 125(1), 232-237.
2. CTs have been associated also to deep pressure touch – which is supposedly is involved in handholding: Case, L. K., Liljencrantz, J., McCall, M. V., Bradson, M., Necaise, A., Tubbs, J., ... & Bushnell, M. C. (2021). Pleasant deep pressure: expanding the social touch hypothesis. Neuroscience, 464, 3-11.
3. There are studies already showing individual preferences for the types of touch (see for example, Perini, I., Olausson, H., & Morrison, I. (2015). Seeking pleasant touch: neural correlates of behavioral preferences for skin stroking. Frontiers in behavioral neuroscience, 9, 8.)**

The possibility for CT effects in handholding is an exciting new development; however, such effects have not been directly demonstrated. The density of CTs in non-hairy skin is still much lower than on hairy skin, suggesting that even if they occur, these effects are likely to be much smaller than with stroking, and as such do not provide an explanation for the preference for handholding found in our study. Still, we mention this new development in a footnote:

*Recent studies have found some C-tactile afferents in the palm skin as well, albeit at a much lower density [12]; other studies show that these afferent can be activated by deep pressure and not only be stroking, which might mean that they are activated by handholding as well [13]. As such, we avoid using the term C-touch for stroking in the rest of the manuscript as both types of touch might be activating C-tactile afferents.*

The study by Perini and colleagues is reviewed with multiple similar studies in a meta-analysis by Cruciani and colleagues (2021), which we already cite.

Cruciani, G., Zanini, L., Russo, V., Boccardi, E., & Spitoni, G. F. (2021). Pleasantness ratings in response to affective touch across hairy and glabrous skin: a meta-analysis. *Neuroscience & Biobehavioral Reviews*, *131*, 88-95.

**• Page 6: When citing the work of Reddan and colleagues, I think it is very important and informative to also cite the (several) publications from Charlotte Krahé and Aikaterini Fotopoulou that have investigated the role of touch in modulating pain (I have mentioned them in one of my points above).**

The work of Reddan and colleagues is mentioned as one of the only effectiveness studies comparing handholding and stroking. The publications the reviewer noted above compare stroking of the forearm with other controls (e.g., stroking of the forearm at different velocities). As detailed above, we now cite most of the studies by Krahé and Fotopoulou earlier in the manuscript when discussing evidence for the effectiveness of stroking, but they do not address the comparison between handholding and stroking.  **• Page 7: “While research on the effectiveness of these types of touch has achieved mixed results…” What are these mixed results the Authors are referring too? I do not thing that have been discussed properly. What are the controversies/open questions/outstanding issues in this field? This is unclear and should be discussed here.**

We appreciate this comment, as this sentence was indeed unclear. We now clarify that mixed results refer to research comparing the effectiveness of handholding and stroking, of which we know only of the study by Reddan and colleagues which found null results:

*While research comparing the effectiveness of handholding and stroking has achieved mixed results, no study has directly compared subjective preferences…*

We now state clearly the open question we are addressing:

*While effectiveness studies attempt to explore how people experience various types of touch, they do not examine which touch people actually prefer or choose to provide, when they encounter the need to regulate emotions in real-life. Touch choice may be based on representations of touch effectiveness, but it might also have other considerations. For example, people could be choosing types of touch they have seen others perform or which were depicted culturally (i.e., in various forms of media), or types of touch which require less physical effort. Thus, effectiveness studies are not enough to understand which types of touch people choose to use. The current study aims to address this gap in the literature by examining subjective preference - presenting people with various situations and asking them which types of touch they would prefer to receive or to provide.*

**• Page 8: I think this paper would be very relevant here, and I am very surprised it has not been mentioned at all:

von Mohr, M., Kirsch, L. P., & Fotopoulou, A. (2017). The soothing function of touch: affective touch reduces feelings of social exclusion. Scientific reports, 7(1), 1-9.**

The paper is now mentioned as part of the paragraph detailing studies showing the effectiveness of stroking.

**• Page 8: “We did not have any hypothesis concerning valence and physicality”. It is very hard to understand what the Authors mean with valence. I suggest including some examples in parathesis to explain what you mean by “valence”. Furthermore, I am very surprised that no hypothesis was formulated about valence. Surely one would expect that a positive or a negative emotion would make a difference (if I understand what the Authors mean by valence, as it has not been spelled out)…**

We now detail what we mean by valence:

*(i.e., whether the experience is positive or negative)*

Regarding an hypothesis, we shared the reviewer’s opinion that valence would make a difference but did not find theoretical support for any specific direction (e.g. that one of the types of touch would be more strongly preferred than the other in positive, as opposed to negative, situations). We have slightly adjusted the sentence to state that:

*We did not have a specific directional hypothesis concerning valence and physicality.*

Interestingly, the data did not support our (and the reviewer’s) notion that valence should make a difference, except for the fact that both types of touch were rated higher in negative, as compared to positive, situations.

**• Page 8: “…we hypothesized that the preference for handholding would be stronger in intense situations, involving strong, acute emotions”. Again, this is very unclear. What do you mean by “strong, acute emotions”?**

This was a stylistic choice. As it may have been confusing, we have removed this wording. This section of the paragraph now reads:

*Regarding intensity, the homeostatic regulation model led us to believe that if two-way sensory communication is indeed crucial for regulating acute disturbances in homeostasis, handholding may be more effective, and therefore preferred, as a way to regulate emotions in intense situations. Thus, we hypothesized that the preference for handholding would be stronger in intense situations.*

**• Page 9: Why the Authors did choose Arabic-speaking and Hebrew-speaking participants to investigate cultural differences? This implies some differences in emotion regulation or in the use of touch due to the spoken language, but this is what I imagine, as it has not been explained, contextualized, rationalized here.**

We wish to thank the reviewer for this comment, as our choice certainly requires more explanation. In an Israeli context, in which the study was conducted, the Jewish and Arab ethnic groups strongly differ in their language proficiencies, to the extent that it is common practice when comparing these group to use language as a proxy for culture. We now discuss this in the manuscript:

*In Israel, where the study was performed, Hebrew native speakers are overwhelmingly Jewish while Arabic native speakers are overwhelmingly Arab [41], making the use of language as a proxy for culture a common practice in research on these cultural groups (e.g., [42,43]).*

**• Page 10, Open data and preregistration: I must say that this is the first time I see the word “videos” so far and it came a bit of nowhere. I think that the fact that the experiments are done online, and videos are used should be introduced much earlier in the manuscript (e.g., introduction). Readers go through the hypotheses without knowing a very important detail about the used approach. There are several studies that used videos of touch, and this opens the door to a field of studies (sometime referred to as vicarious touch) that has not been mentioned at all in the introduction and I think it is of crucial importance here. Participants are not simply imagining of being touched (as I originally thought) but they are watching videos of people touching. I strongly encourage the Authors to consider adding some references from the vicarious touch relevant and introduce this topic in the introduction.**

We now mention videos in the abstract:

In four pre-registered online studies (total N=287), participants rated handholding and stroking, presented in short videos, as emotion regulation methods

And in a full paragraph in the introduction, which also addresses vicarious touch:

***Optimal stroking and the use of videos***

*As detailed above, the aim of the current study was to connect effectiveness research with subjective preferences. While many types of stroking exist, stroking researchers have focused on gentle stroking of the forearm by the hand at a velocity of 3 cm/s as the optimal stroking speed; in fact, stroking at higher speeds is often used as a control condition (e.g., in a study by von Mohr and colleagues [18]). As our hypothesis was that handholding would be preferred over stroking, we sought to present stroking in the optimal way possible so that our design tests the hypothesis effectively.*

*However, this presented a challenge. Asking people to rate “stroking” might lead them to rate suboptimal forms of stroking, artificially deflating ratings of stroking. On the other hand, asking them to rate “stroking of the forearm at a 3 cm/s speed”, when compared to “handholding”, might feel complex or artificial. We used two strategies to overcome this issue. In most of the presented studies, we used looping videos instead of verbal labels for the types of touch. In Study 4, we asked participants which types of touch were provided to them during childbirth and provided standard verbal labels – “stroking” and “handholding”. As both optimal and non-optimal forms of stroking would count towards “stroking” in this paradigm, we could be confident that the prevalence of optimal stroking would, if anything, be overestimated.*

*Notably, the use of videos somewhat resembles paradigms involving vicarious touch – the study of the effects of seeing people touching one another [29]. However, the current study does not ask people about their feelings while watching the videos, or their thoughts about the videos or about the people depicted in them, which are the questions explored by vicarious touch research. Instead, the current study asks participants about hypothetical or recalled scenarios and only uses the videos to describe the types of touch. Of course, some vicarious effects might be triggered by watching the videos, but the same is true for actual touch in real life – while touching or being touched by another person, we usually also see the act of touching. Additionally, to the best of our knowledge, no study of vicarious touch has compared observing handholding to observing stroking.*

**• Page 17, Statistical analysis: Which software/package was used for data analysis? This information should be added.**

We now cite all of the software and packages used for data analysis. **• Page 25-18, Results: I must say that I found extremely difficult to follow and understand the results. The results are presented in tables with no sufficient explanation, and I think that the main results should also be reported in the text. Furthermore, expressions such as “almost always” “in almost all studies” should be avoided in the results section. I would like to see more precision and clarity. I am wondering if reporting the results study by study rather than organized in topics would be more helpful.**

We have completely reworked the results section, and wish to thank the reviewer for the suggestion to report results study by study. We hope this change improves the clarity of the results. We have also removed the use of phrases such as “almost all”.

**• Page 30, Discussion: I think that it is quite confusing and beyond the point to answer the question “Why is handholding preferred over stroking?” by referring to Greek literature and the story of Adam and Eve. The Authors should stay closer to the data and what it is known in the scientific literature in humans. The discussion overall is too brief and does not help in understanding the results.**

The discussion as a whole has been redesigned – we hope that the rewritten discussion helps address the reviewer’s general concern.

Regarding references to literature, we believe that the fact that literature scholars studying key cultural texts find that handholding is an important theme lends support to our suggestion that some of the effects of handholding might be due to its cultural prevalence. Still, we have significantly reduced our discussion of these issues, in favor of other issues noted by the reviewer in other comments such as expanding our discussion of bottom-up and top-down processes. The sentence now reads:

*However, it could also be the case that the preference for handholding is unrelated to differences in effectiveness. One possibility is that as handholding is more prevalent in society, it is more recognizable and enjoys a familiarity effect[45]. Holding hands is ubiquitous and even appears in some monkey populations [46], and features in a variety of cultures: for example, it is mentioned in well known Western cultural texts [47,48], is a popular public gesture of affection in Vietnamese culture [49], and features in indigenous Taiwanese dances [50].*

**• Page 30-31, it is unclear to me why top-down factors play a more important role in handholding as compared to stroking. There is a huge increase in research showing the impact of intentionality, cognitive factors, and top-down factors in stroking studies, and these would go completely against this explanation. See for example:

Sailer, U., & Leknes, S. (2022). Meaning makes touch affective. Current Opinion in Behavioral Sciences, 44, 101099.
Sailer, U., Hausmann, M., & Croy, I. (2020). Pleasantness only? How sensory and affective attributes describe touch targeting C-tactile fibers. Experimental Psychology, 67(4), 224.
Ellingsen, D. M., Leknes, S., Løseth, G., Wessberg, J., & Olausson, H. (2016). The neurobiology shaping affective touch: expectation, motivation, and meaning in the multisensory context. Frontiers in psychology, 6, 1986.
McCabe, C., Rolls, E. T., Bilderbeck, A., & McGlone, F. (2008). Cognitive influences on the affective representation of touch and the sight of touch in the human brain. Social cognitive and affective neuroscience, 3(2), 97-108.

Furthermore, it is also unclear why the paper of Cascio et al that is cited here would support this view.**

We did not intend to claim that top-down processes play a more important role in handholding compared to stroking, but that in stroking bottom-up processes play a larger part than in handholding. As discussed by Cascio et al.:

*What makes touch “social?” One way of operationalizing social touch has been handily provided by the correspondence between properties of much naturalistic affiliative interpersonal touch and the unique tuning characteristics of low-threshold unmyelinated peripheral afferent fibers (C-touch, or CT fibers).*

They then equate CT afferent activation with bottom-up processes:

*Having considered social touch from the bottom (peripheral afferents)-up, we now turn to top-down contextual factors that influence how social touch is defined.*

Following the reviewer’s comment, we have edited the paragraph discussing this to stress that stroking also involves top-down processes:

*Another possibility is that top-down processes might lead to handholding being thought of as more effective. While, as discussed in the introduction, bottom-up processes such as the activation of CT fibers [50] may be more dominant in stroking, top-down processes have been identified in both types of touch. For example, studies have shown that participants’ relationship satisfaction moderated the effects of handholding, such that participants with more satisfying relationships exhibited stronger emotion regulation effects [9,51]; another study has shown that participants’ attachment style moderated the pleasantness caused by stroking, such that participants with more anxious attachment styles experienced less pleasantness. While we could find no direct comparison of top-down effects, one study [52] explored difference in the ways people perceive reciprocal types of touch, which include handholding, as opposed to non-reciprocal types of touch, which include stroking (although these two specific types of touch were not directly examined in the study). The study showed that people regarded both participants in reciprocal touch slightly higher (on dimensions of valence and likability) than providers of non-reciprocal touch, and significantly higher than recipients of non-reciprocal touch. Thus, people thinking about providing or receiving handholding might be thinking about themselves as more likeable and with a more positive valence, which might lead them to choose this type of touch over others regardless of its actual bottom-up effects.*
